# Deposition of a cutin apoplastic barrier separating seed maternal and zygotic tissues

**DOI:** 10.1186/s12870-019-1877-9

**Published:** 2019-07-10

**Authors:** Olivier Coen, Jing Lu, Wenjia Xu, Delphine De Vos, Christine Péchoux, Frédéric Domergue, Damaris Grain, Loïc Lepiniec, Enrico Magnani

**Affiliations:** 1 Institut Jean-Pierre Bourgin, INRA, AgroParisTech, CNRS, University of Paris-Saclay, Route de St-Cyr (RD10), 78026 Versailles Cedex, France; 2 École Doctorale 567 Sciences du Végétal, University Paris-Sud, University of Paris-Saclay, bat 360, 91405 Orsay Cedex, France; 3 INRA, Génétique Animale et Biologie Intégrative, Domaine de Vilvert, Cedex, 78352 Jouy-en-Josas, France; 4 Laboratoire de Biogenèse Membranaire, University of Bordeaux, UMR 5200, CNRS /, 71 av. E. Bourleaux, CS 20032, 33140 Villenave d’Ornon, France

**Keywords:** Seed coat, Cuticle, Fertilization

## Abstract

**Background:**

In flowering plants, proper seed development is achieved through the constant interplay of fertilization products, embryo and endosperm, and maternal tissues. Communication between these compartments is supposed to be tightly regulated at their interfaces. Here, we characterize the deposition pattern of an apoplastic lipid barrier between the maternal inner integument and fertilization products in *Arabidopsis thaliana* seeds.

**Results:**

We demonstrate that an apoplastic lipid barrier is first deposited by the ovule inner integument and undergoes de novo cutin deposition following central cell fertilization and relief of the FERTILIZATION INDEPENDENT SEED Polycomb group repressive mechanism. In addition, we show that the WIP zinc-finger TRANSPARENT TESTA 1 and the MADS-Box TRANSPARENT TESTA 16 transcription factors act maternally to promote its deposition by regulating cuticle biosynthetic pathways. Finally, mutant analyses indicate that this apoplastic barrier allows correct embryo sliding along the seed coat.

**Conclusions:**

Our results revealed that the deposition of a cutin apoplastic barrier between seed maternal and zygotic tissues is part of the seed coat developmental program.

**Electronic supplementary material:**

The online version of this article (10.1186/s12870-019-1877-9) contains supplementary material, which is available to authorized users.

## Background

In the course of evolution, land plants have developed hydrophobic apoplastic interfaces to prevent water loss, gain protection against biotic and abiotic stresses, establish diffusion barriers and prevent organ fusion [44, 65]. The most widespread of these barriers is the cuticle, an external hydrophobic layer that covers epidermal cell walls of aerial organs. Cutin is the insoluble component of cuticles and is composed of an amorphous, cross-linked matrix of lipid polyesters bound to the cell wall [44, 65]. Waxes, mainly composed of very-long-chain fatty acid (VLCFA) derivatives, are often embedded within this polyester matrix, or deposited onto its outer surface [44, 65]. By contrast, suberin layers, the other known apoplastic interfaces, are deposited on the inner face of primary cell walls [44, 60]. Suberin layers act as hydrophobic barriers in endodermal and peridermal cells, where they enable nutrient-selected uptake by forcing solutes to transit through symplastic connections [60]. The composition and ultrastructure of cutin and suberin vary considerably according to the species, organ and developmental stage [44, 65]. Nevertheless, cutin and suberin found in seed plants are generally made in large part of C16 and C18 hydroxy alkanoic acids and their derivatives. In most species, hydroxy fatty acids are typically found in cutin, whereas dicarboxylic fatty acids (DCA) are considered specific to suberin [26, 44, 65]. Nonetheless, in *Arabidospsis thaliana,* stem and leaf cuticles were found to contain 52 to 60% of C18:2 DCA, which was not detected in root suberin [37]. This compound is therefore considered a hallmark of cutin in Arabidopsis.

A number of genes have been discovered to be involved in cuticle deposition in Arabidopsis. Some of them encode key enzymes in cutin biosynthesis, such as *ABERRANT INDUCTION OF TYPE THREE 1* (*ATT1*), *BODYGUARD* (*BDG*), *DEFECTIVE IN CUTICULAR RIDGES* (*DCR*), *LONG-CHAIN ACYL-COA SYNTHETASE 2* (*LACS2*), *GLYCEROL-3-PHOSPHATE SN-2-ACYLTRANSFERASE 4/8* (*GPAT4/8*) and *FATTY ACYL-ACP THIOESTERASES B* (*FATB*) [34, 36, 42, 48, 55]. Other genes, such as *ECERIFERUM 6* (*CER6*) and *3-KETOACYL-COA SYNTHASE 1* (*KCS1*), are involved in VLCFA biosynthesis, while *CER1* is required for waxes (i.e. alkanes) production [7, 41, 57]. Besides, *WHITE-BROWN COMPLEX HOMOLOG PROTEIN 11* (*WBC11*) and *CER5*, encode ABC transporters that play a role in the apoplastic export of cutin and wax monomers, respectively [49, 59]. Finally, a handful of transcription factors were shown to tightly regulate cutin and wax biosynthesis. For instance, among AP2/EREBP family members, WRINKLED (WRI) 1, 3 and 4, and SHINE (SHN)/WAX INDUCER (WIN) 1, 2 and 3 positively regulate cutin accumulation [32, 47, 56], whilst DEWAX 1 and 2 repress wax biosynthesis [29, 33]. Several MYB transcription factors, such as MYB16, MYB30, MYB94, MYB96 and MYB106, also play crucial roles in these processes [35, 46, 47, 50]. In particular, MYB30 was shown to regulate genes involved in wax as well as cutin biosynthesis [50].

Apoplastic barriers have been speculated to play a crucial role in seed development. In angiosperms, seeds are composed of three genetically distinct components: embryo, endosperm, maternal tissues (encompassing funiculus, chalaza, nucellus and seed coat) [31]. To achieve proper seed development, from fertilization to maturation, maternal tissues and fertilization products need to grow in a coordinated fashion by constantly communicating through molecular and mechanical signaling [23, 28, 31, 63]. The interfaces that separate these different seed components might therefore play a role in such delicate tissue cross-talk. The embryo cuticle resembles the one covering aerial tissues and acts as a first interface between embryo and endosperm, in addition to the embryo sheath [43, 62]. Moreover, other apoplastic barriers were reported in seed maternal tissues. Creff and coworkers showed a discontinuous electron-dense apoplastic layer in between inner and outer integuments in developing seeds [13]. It was also shown that suberin seals the hilum of mature Arabidopsis seeds [5, 17]. Finally, several studies have reported an electron-dense lipid apoplastic barrier at the interface between the endosperm and the seed coat, in developing seeds as well as in mature seeds [4, 14, 17, 38]. Loubéry and coworkers have demonstrated that such an apoplastic barrier, laying on the adaxial side of the endothelium, is of maternal origin and its deposition and integrity is regulated by TRANSPARENT TESTA (TT) transcription factors [38].

The role of the apoplastic barrier in between the seed coat and the endosperm is of special interest as both tissues drive seed growth after fertilization [22, 23, 52]. The Arabidopsis seed coat comprises an inner and an outer integument [12]. Integuments originate from the ovule chalazal tissue as primordia. Later on, both integuments grow by anticlinal cell divisions to progressively surround the nucellus. Interestingly, the adaxial cell layer of the inner integument, termed inner integument 1 (ii1) or endothelium, finds itself enclosed at the very inside of the ovule, in spite of being of dermal origin. After fertilization, the endosperm triggers integument growth and differentiation into the seed coat [22, 23]. Genetic screens have been carried out for mutant seeds deficient in proanthocyanidin (PA) accumulation, a hallmark of endothelium differentiation [16], and revealed *TT1* and *16,* both expressed in the endothelium, as positive regulators of endothelium development and differentiation. *TT16* encodes a MADS-Box transcription factor necessary for proper polar development and cell architecture of the endothelium and its adjacent parenchymatic cell layer, the ii1’ [11, 21, 24, 45]. Furthermore, *TT16* promotes nucellus degeneration after fertilization [63]. *TT1*, which encodes a WIP zinc-finger transcription factor, has also been shown to affect endothelium cell shape but its function in seed coat development remains elusive [2, 53]. The *tt1* mutation affects the deposition of the endothelium apoplastic barrier and both *tt1* and *tt16* seeds display high permeability to toluidine blue, a marker of cuticular permeability [38]. Despite recent advances in the study of endothelium development, little is known on the composition and deposition pattern of the apoplastic barrier covering the endothelium.

Here, we present a thorough analysis of the maternal apoplastic barriers in Arabidopsis ovules and seeds. We show that the apoplastic barrier in between the endothelium and the endosperm is mainly composed of cutin and that de novo cutin deposition occurs after fertilization under the control of the FERTILIZATION INDEPENDENT SEED Polycomb group repressive mechanism. Furthermore, we show that TT16 and TT1 act maternally to promote its deposition and regulate the expression of key genes involved in cuticle biosynthesis. Finally, our findings demonstrate that such an apoplastic barrier is of maternal origin and suggest a role in proper embryo development.

## Results

### Deposition of apoplastic lipid barriers in wild type ovule and seed maternal tissues

**Fig. 1:**
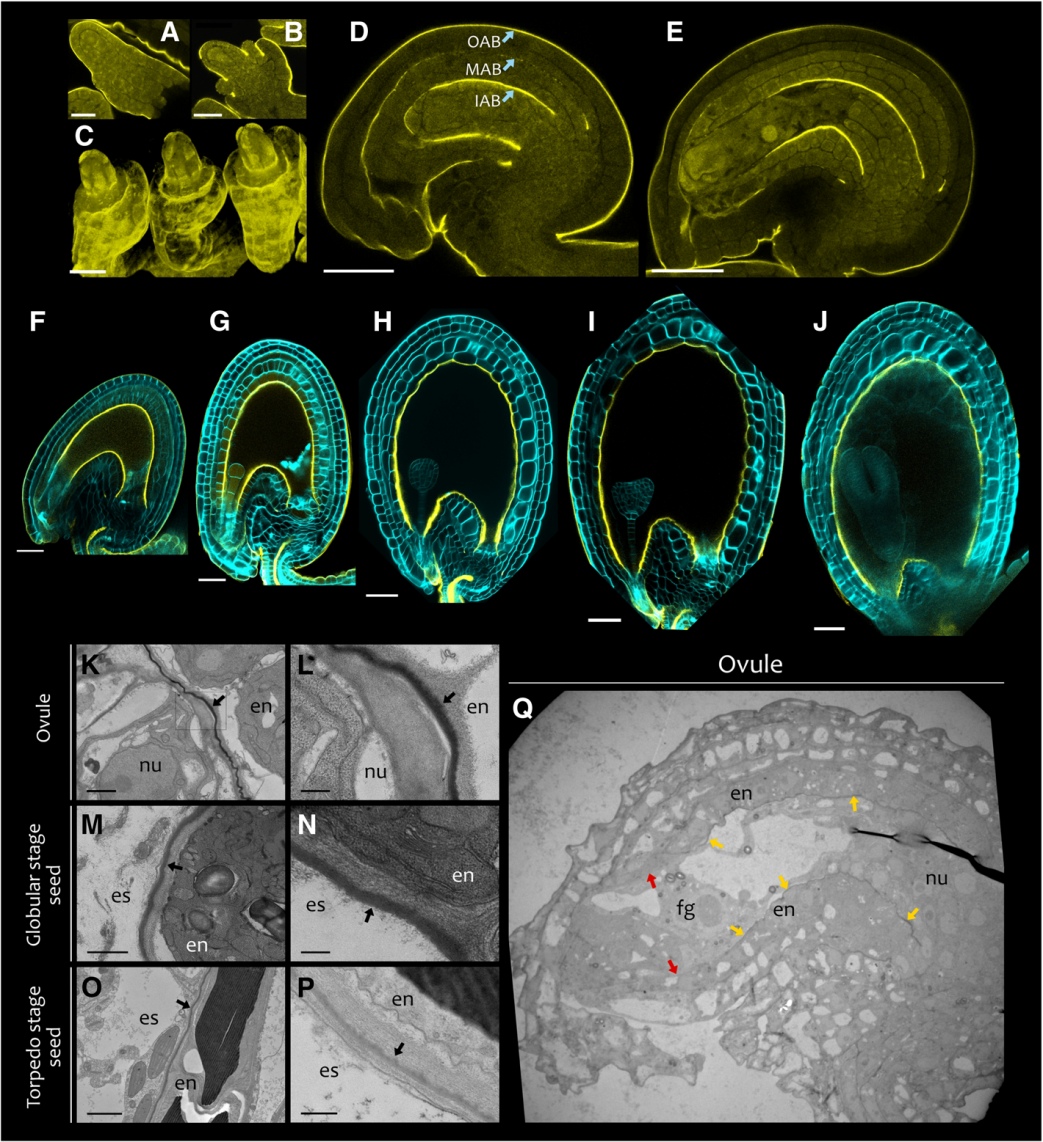
Deposition pattern of apoplastic barriers in ovule and seed integuments. **a**, **b**, **d**, and **e** Fluorescence images of longitudinal sections of wild type ovules at stages (**a**) 2-i, (**b**) 2-iv, (**d**) 3-iv and (**e**) 3-VI [54], stained with auramine O (yellow). **c** Fluorescence image of a three-dimensional reconstruction of a wild type ovule at stage 2-IV stained with auramine O (yellow). **f** to **j** Fluorescence images of longitudinal sections of wild type seeds at different developmental stages stained with auramine O (yellow). Seed cell walls are counterstained with calcofluor (cyan). **k** to **q** Transmission electron micrographs showing the IAB of wild type ovules and seeds. **k** Mature ovule (stage 3-VI). **l** Close-up image of (**k**). **m** and **n** Globular embryo stage seed. **o** and **p** Torpedo embryo stage seed. **q** Mature ovule (stage 3-VI). Black arrows indicate the IAB. Yellow arrows indicate the presence of the IAB between endothelium and nucellus/female gametophyte, whereas red arrows its absence. en, endothelium; nu, nucellus; es, endosperm; fg, female gametophyte. Ecotype Col. Scale bars: (**a**) 10 μm, (**b**) and (**c**) 20 μm, (**d**) and (**e**) 30 μm, (**f**) to (**j**) 50 μm, (**k**) and (**m**) 1 μm, (**o**) 2 μm, (**l**), (**n**) and (**p**) 0.25 μm, (**q**) 10 μm.

Arabidopsis seeds displayed a birefringent layer in between the seed coat and the endosperm when analyzed using Differential Interference Contrast (DIC) microscopy (Additional file 1: Figure S1A). Likewise, we observed an auto-fluorescent layer at the exact same position by confocal microscopy (Additional file 1: Figure S1B and C). To test if such apoplastic layers are of lipidic nature, we stained wild type ovules and seeds with auramine O, a fluorescent dye used to detect plant cuticles [9]. Auramine O fluorescence revealed apoplastic lipid layers covering the integuments and the nucellus of ovule primordia (Fig. 1). Young primordia displayed spotty fluorescence, especially at the distal tip of inner integuments, thus suggesting that the deposition of such lipid layers happens in a discontinuous manner (Fig. 1a and b). At stage 3-IV of ovule development [54], when integuments fully cover the nucellus, auramine O marked three apoplastic laminar structures: a first layer in between the nucellus and the endothelium, a second layer in between the inner and the outer integument, and a third layer covering the abaxial (outer) side of the outer integument, thereafter referred to as Inner (IAB), Middle (MAB), and Outer (OAB) Apoplastic Barriers, respectively (Fig. 1d). Later in ovule development, the distal micropylar region of the IAB and the MAB were almost undetectable by auramine O staining (Fig. 1e).

After fertilization, we still observed intense IAB fluorescence separating the endothelium from the nucellus and the newly formed endosperm (Fig. 1f to j), as previously observed [38]. Whereas the IAB signal remained strong in distal and proximal regions, it appeared to slightly diminish in the intervening curving zone during seed development (Fig. 1f to j). In line with previous results, the distal micropylar region of the seed did not stain with auramine O, at all stages analyzed (Fig. 1f to j) [38]. Furthermore, the MAB was either faintly fluorescent or undetectable at globular embryo stage and undetectable by heart embryo stage onward (Fig. 1f to j). The seed OAB had the tendency to detach, probably due to the clearing process (Fig. 3j), and therefore we could not reliably follow its development by auramine O staining. All these observations were independent of the Arabidopsis ecotype tested (Col, Ws or Ler) (Additional file 1: Figure S2).

To better characterize the structure of these apoplastic barriers and confirm auramine O staining analyses, we studied wild type ovules and seeds by Transmission Electron Microscopy (TEM). The ovule IAB appeared as a thick, dark electron-dense layer in between nucellus and endothelium cell walls (Fig. 1k and l), typical of cuticle layers [44, 65]. The occasional splitting of the IAB into two layers, a more electron-dense one on the endothelium side and a fainter one on the nucellus side, revealed that the ovule IAB originates to a wider extent from the endothelium (Additional file 1 Figure S3). The distal micropylar region of the IAB, enclosing the female gametophyte, appeared thinner than in more proximal regions (Fig. 1q). Overall, ovules exhibited a decreasing gradient in IAB thickness along the proximal–distal axis (Fig. 1q). Furthermore, we observed discontinuities in the MAB (Additional file 1: Figure S4).

After fertilization, at globular embryo stage, we detected a less electron-dense IAB separating endothelium and endosperm, when compared to ovules (Fig. 1m and n). At torpedo embryo stage, the IAB was still present but appeared even less electron-dense compared to earlier stages (Fig. 1o and p).

### De novo deposition of cutin in the IAB after fertilization

**Fig. 2:**
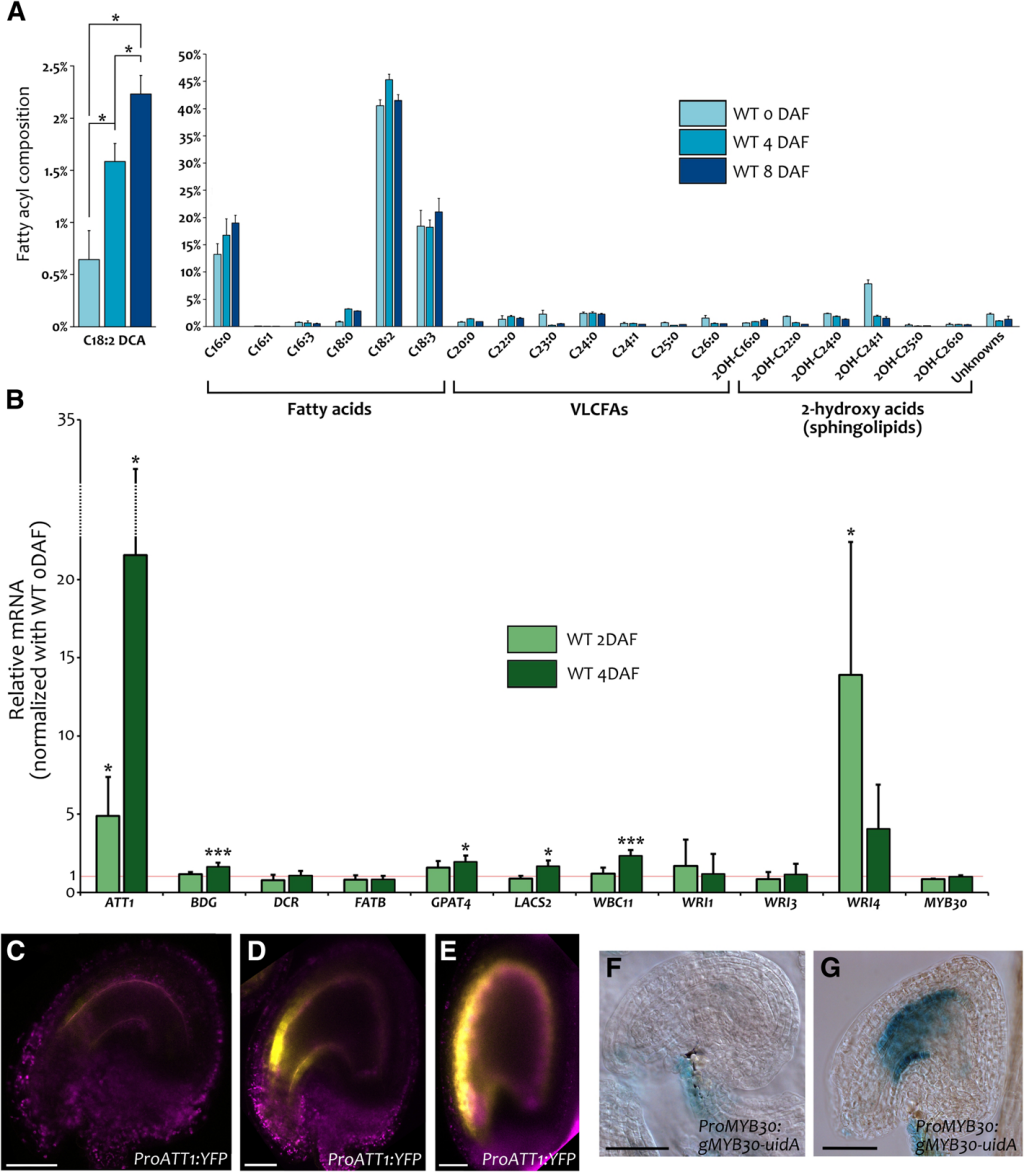
IAB de novo cutin deposition after fertilization. **a** Analyses of ovule (0 DAF) and seed (4 and 8 DAF) fatty acyl composition by GC-MS. Values are relative to all detected fatty acyl chains. **b** RT-qPCR analyses of genes involved in cutin deposition in wild type seeds at 2 and 4 DAF. Values are relative to wild type ovules at 0 DAF. **c** to **e** YFP fluorescence (yellow) and auto-fluorescence (purple) images of *ProATT1:YFP* ovules (stage 3-VI) (**c**) and seeds at 2 cells (**d**) and late globular (**e**) embryo stages. **f** and **g** GUS activity in cleared whole mounts of a *ProMYB30:gMYB30-uidA* ovule (stage 3-VI) (**f**) and seed right after fertilization (**g**). WT, wild type. Error bars represent standard deviations. Asterisks indicate statistical difference between different time points (Student’s t test, *: *P* < 0.05; **: *P* < 0.01; ***: *P* < 0.001). The complete statistical analysis of (**a**) is in Additional file 1: Table S3. Ecotype Col. Scale bars: (**c**), (**d**), (**f**) and (**g**) 30 μm, (**e**) 50 μm

The sizeable increase in IAB surface following fertilization raises the question of de novo IAB deposition during seed development. To test this hypothesis, we compared the fatty acyl composition of wild type ovules at 0 Days After Flowering (0 DAF) and seeds (4 DAF and 8 DAF) by Gas Chromatography-Mass Spectrometry (GC-MS). At 4 DAF, the embryo cuticle is not fully formed yet [30], thus allowing us to test the lipid composition of the seed coat apoplastic barriers. We observed significant changes in the composition of several compounds (fatty acids, VLCFAs and 2-hydroxy acids of sphingolipids) associated with cell membranes (Fig. 2). Nonetheless, we did not detect VLCFA derivatives. The relative content of C18:2 DCA was increased by more than 2.6 folds from 0 to 4 DAF, and more than 3.7 folds from 0 to 8 DAF (Fig. 2a). Given that C18:2 DCA is considered a good marker of cutin in Arabidopsis, these results indicate a significant and progressive de novo deposition of cutin in seeds after fertilization.

We then aimed at determining whether seed cutin deposition is associated with changes in gene expression. To this end, we chose a set of cutin-related genes, predicted to be expressed in the seed coat with an average signal intensity higher than 500 according to Belmonte and coworkers’ microarray data [6], and compared their expression levels by RT-qPCR in ovules (0 DAF) and seeds (2 and 4 DAF). *ATT1*, *BDG, GPAT4, LACS2*, *WBC11* and *WRI4* showed a significant upregulation after fertilization (Fig. 2b). In particular, the expression of *ATT1,* a gene directly involved in C18:2 DCA deposition, raised by 5 folds at 2 DAF and 20 folds at 4 DAF (Fig. 2b), compared to 0 DAF. Likewise, fertilization marked the change in expression of a set of genes involved in VLCFA (Additional file 1: Figure S5). Overall, these data are in line with the microarray data of Figueiredo and coworkers’s, which compare the transcriptomes of unfertilized ovules 4 Days After Emasculation (DAE), and fertilized seeds 2 Days After Pollination (DAP) [22]. The expression of *ATT1*, *GPAT4*, *BDG*, *LACS2*, *DCR* and *WBC11* cutin-related genes increased from 1.9 to 6.4 folds in fertilized seeds compared to unfertilized ovules.

The activity of the *ATT1* promoter region in seed has been shown to be endothelium-specific [42]. To better characterize *ATT1* expression pattern and correlate it to our expression data, we thoroughly analyzed the activity of the *ATT1* promoter region fused to *YFP* in ovules and seeds. In mature ovules, fluorescence was barely detectable in a small region of the endothelium near the micropyle (Fig. 2c). By contrast, a stronger signal was observed in the endothelium after fertilization, confirming our RT-qPCR expression analyses (Fig. 2d and e). Fluorescence was first localized in the distal region of the endothelium, and then expanded to the whole endothelium. The activity pattern of the *ATT1* promoter region strongly suggests that the raise in C18:2 DCA observed after fertilization is the result of de novo cutin deposition in the IAB, likely occurring in a distal-proximal manner.

Since *MYB30* is involved in both cutin and VLCFA deposition and is expressed in the seed coat [6], we characterized its expression pattern before and after fertilization. We created a marker line carrying *MYB30* 2.1 kb promoter region and coding sequence, translationally fused to *uidA* (encoding the β-glucuronidase protein, GUS). Whereas we observed GUS staining solely in the funiculus of mature ovules (Fig. 2f), three independent *ProMYB30:gMYB30-uidA* lines displayed staining both in endothelium and funiculus of seeds at early globular embryo stage (Fig. 2g). We therefore speculate that *MYB30* might also play a role in de novo cutin deposition after fertilization.

### *TT16* and *TT1* promote IAB deposition

**Fig. 3:**
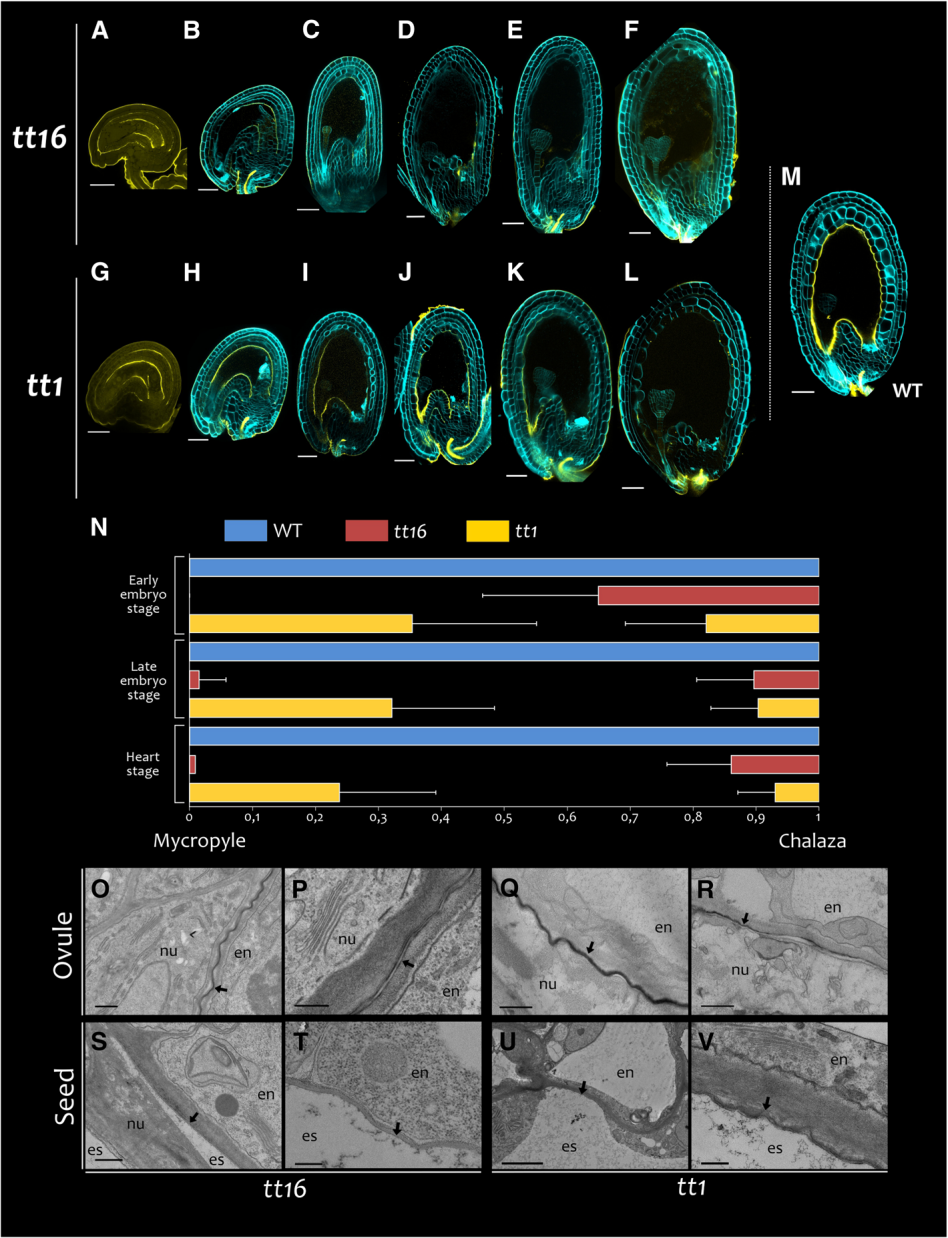
TT16 and TT1 promote IAB deposition. **a** to **m** Fluorescence images of longitudinal sections of representative *tt16* (**a** to **f**), *tt1* (**g** to **l**) and wild type (**m**) ovules and seeds stained with auramine O (yellow). Seed cell walls are counterstained with calcofluor (cyan). **n** Position of detectable auramine O signal in wild type, *tt16* and *tt1* seeds along the distal-proximal (micropyle-chalaza) axis. The micropyle-chalaza axis was assimilated to a [0,1] segment (see methods). Error bars represent standard deviations, *n* > 10. **o** to **v** Transmission electron micrographs showing the IAB of *tt16* and *tt1* ovules (stage 3-VI) and seeds at globular embryo stage. Black arrows indicate the interface between endothelium and nucellus/endosperm. en, endothelium; nu, nucellus; es, endosperm. Ecotype Col. Scale bars: (**a**), (**b**), (**g**) and (**h**) 30 μm, (**c**) to (**f**) and (**i**) to (**m**) 50 μm, (**s**) 1 μm, (**p**), (**s**), (**t**), (**u**) and (**v**) 0,25 μm, (**q**) and (**r**) 0,5 μm

Because both *ATT1* and *MYB30* are strongly induced in the endothelium after fertilization, we investigated whether transcription factors responsible for endothelium cell identity, such as TT16, TT1, TTG1 and TTG2, could regulate IAB deposition. The IAB of *ttg1–1* and *ttg2–3* mutant seeds was undistinguishable from that of wild type seeds (Additional file 1: Figure S6). By contrast, auramine O staining was severely affected in both *tt16–1* and *tt1–3* mutant seeds (Fig. 3). At anthesis, some *tt16* ovules displayed reduced auramine O staining compared to wild type (Fig. 3a, compared to Fig. 1e). By contrast, staining appeared wild-type-looking in all analyzed *tt1* ovules (Fig. 3g). After fertilization, the IAB was barely detectable in *tt16* seeds (Fig. 3b to f) and appeared restricted to specific regions in *tt1* seeds (Fig. 3h to l). Comparable defects in auramine O staining were also observed in *tt16–2* and *tt1–4* mutant alleles (Additional file 1: Figure S6E to 5G). By contrast, MAB auramine O staining was not affected by any of the *tt* mutations tested.

Fluorescence of variable intensity was still detectable until heart embryo stage in *tt16* and *tt1* seeds, albeit with different patterns. In order to better characterize such differences, we measured the position of the regions that showed detectable, even if faint, auramine O staining in wild type, *tt16* and *tt1* seeds, at early globular, late globular and heart embryo stages (Fig. 3n; see Methods). In *tt16* seeds, fluorescence was detected almost exclusively in the proximal region from early globular embryo stage onwards, whereas *tt1* seeds displayed staining in the proximal and distal regions, but not in the curving zone.

Consistent with these results, TEM analyses of *tt16* ovules displayed zones with a discontinuous electron-dense IAB, especially in the proximal region, together with zones exhibiting a thinner and less dense IAB compared to wild type (Fig. 3o and p). The IAB of *tt1* ovules was as thick as in wild type but exhibited discontinuities as well (Fig. 3q and r). At globular embryo stage, we did not detect a clear IAB in the proximal and distal regions of *tt16* seeds (Fig. 3s and t) and in the curving zone of *tt1* seeds (Fig. 3u and v).

We conclude from these observations that *TT16* and *TT1* are both essential for correct IAB deposition in seeds, although they fulfill different functions along the proximal-distal axis.

### *TT16* and *TT1* regulate cuticle biosynthetic pathways

**Fig. 4:**
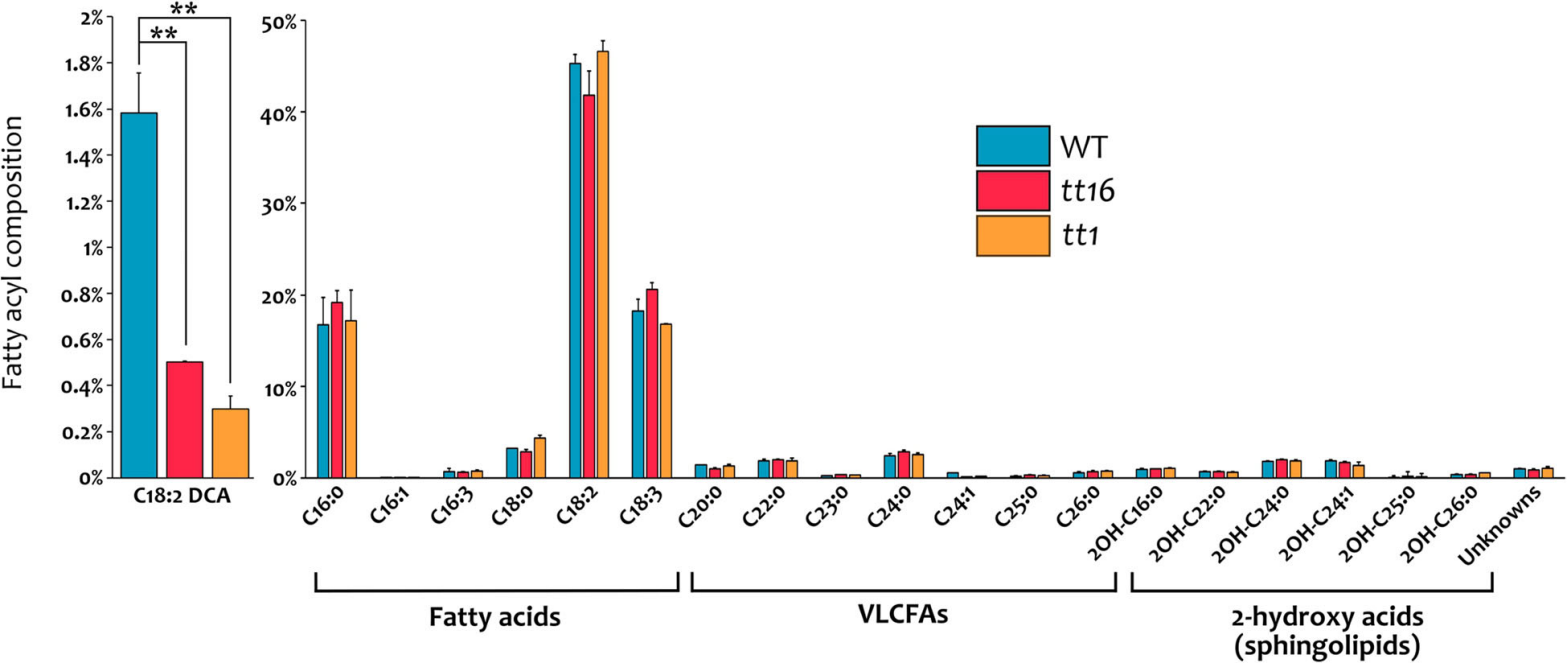
*tt16* and *tt1* seeds display an altered C18:2 DCA composition. Analyses of fatty acyl composition in wild type, *tt16* and *tt1* seeds at 4 DAF by GC-MS. Values are relative to all detected fatty acyl chains. Error bars represent standard deviations. Asterisks indicate statistical difference between different genotypes (Student’s t test, *: P < 0.05; **: P < 0.01; ***: P < 0.001). Ecotype Col. The complete statistical analysis is in Additional file 1: Table S3

To characterize *tt16* and *tt1* phenotypes at the biochemical level, we analyzed and compared the fatty acyl composition of wild type, *tt16* and *tt1* seeds by GC-MS. Whereas cell wall compounds did not display striking differences, the C18:2 DCA content was significantly lower in *tt16* and in *tt1* seeds compared to wild type (Fig. 4, biological replica in Additional file 1: Figure S7). These data indicate that the defects in IAB deposition observed in *tt16* and *tt1* seeds are both associated with a strong reduction in C18:2 DCA.

**Fig. 5:**
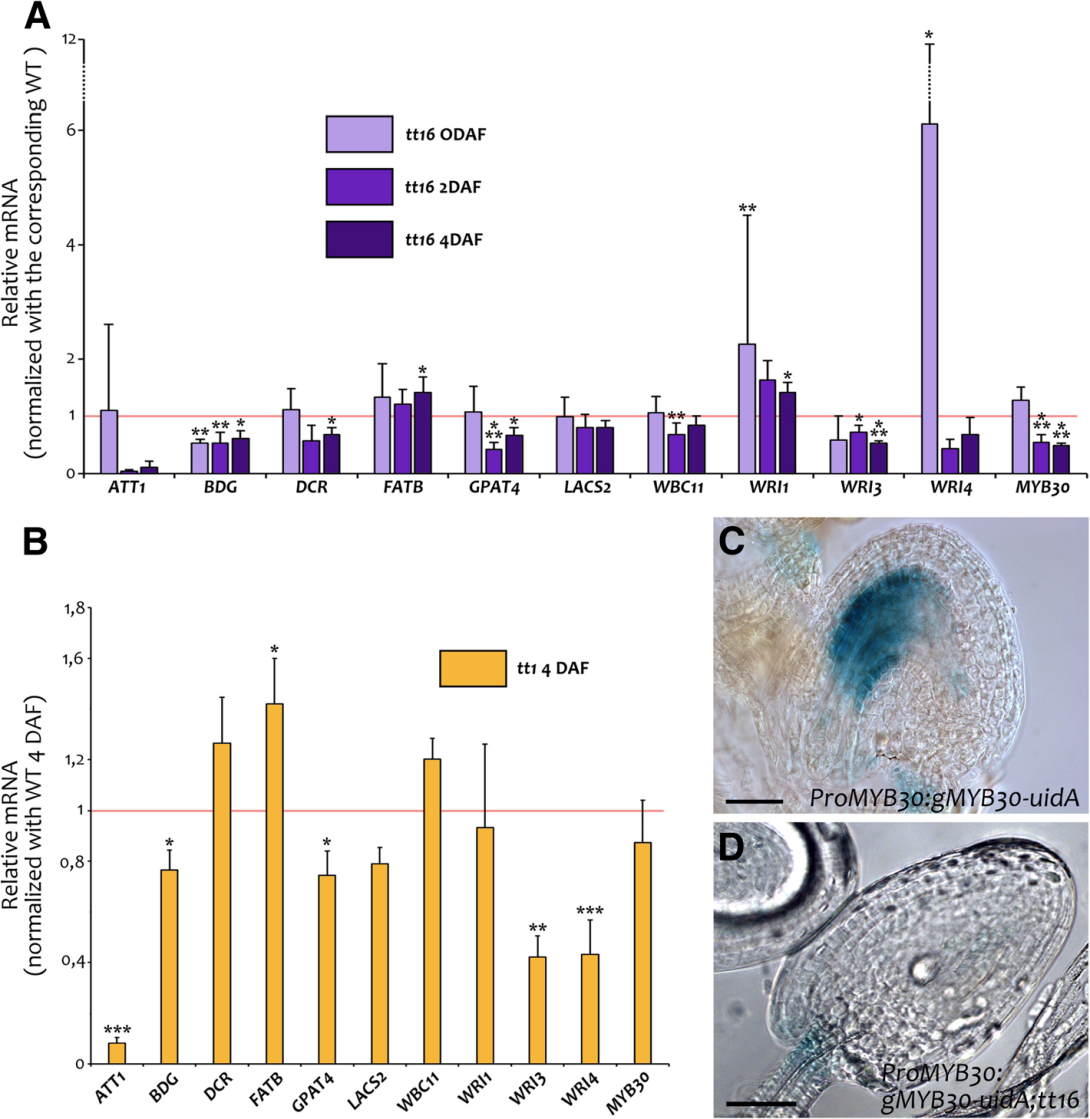
Expression of cutin-related genes in *tt16* and *tt1* seeds. **a** and **b** RT-qPCR analyses of a set of genes involved in cutin deposition in *tt16* ovules (0 DAF) and seeds (2 and 4 DAF) (**a**) and *tt1*seeds (4 DAF) (**b**). Values are relative to wild type. Error bars represent standard deviations. Asterisks indicate statistical difference between mutant and wild type at the same time point (Student’s t test, *: P < 0.05; **: P < 0.01; ***: P < 0.001). Student’s t test for *ATT1* in *tt16* background at 4DAF: *P* = 0.0652. **c** and **d** GUS activity in cleared whole mounts of *ProMYB30:gMYB30-uidA* (**c**) and *ProMYB30:gMYB30-uidA*;*tt16* (**d**) seeds at two-cell embryo stage. Ecotype Col. Scale bars: 30 μm

We investigated whether this drastic decrease in C18:2 DCA correlates with changes in the expression of genes responsible for cutin deposition. To this end, we tested the same set of genes described above (Fig. 2a) in wild type and *tt16* ovules (0 DAF) and seeds (2 and 4 DAF) (Fig. 5). The analysis of such genes in the *tt1* mutant background was limited to seeds at 4 DAF (Fig. 5) as IAB deposition was not drastically affected in *tt1* ovules. In *tt16* ovules, we observed up-regulation of *WRI1* and *WRI4* and down-regulation of *BDG* expression, compared to wild type (Fig. 5a). *ATT1, BDG, GPAT4*, and *WRI3* expression was decreased in both *tt16* and *tt1* seeds (Fig. 5a and b). Furthermore, the expression of *DCR*, *WBC11*, and *MYB30* was reduced in *tt16* seeds (Fig. 5a), whereas *tt1* seeds displayed down-regulation of *WRI4* expression (Fig. 5b). Finally, we observed altered expression of genes involved in VLCFA deposition in both *tt16* and *tt1* seeds (Additional file 1: Figure S8). Overall, these data show that TT16 and TT1 regulate the expression of a number of genes involved in cutin and VLCFA deposition from enzymes, to transporters and transcriptional regulators.

To further test the role of TT16 in *MYB30* expression, we transformed *tt16* plants with the *ProMYB30:gMYB30-uidA* reporter construct. Three out of eight independent *ProMYB30:gMYB30-uidA;tt16* lines displayed GUS staining in the funiculus but not in the endothelium (Fig. 5c and d). Consistent with this result, the introgression of a *ProMYB30:gMYB30-uidA* line, which showed GUS staining both in wild type endothelium and funiculus (Fig. 2g), in a *tt16* background displayed a staining pattern limited to the funiculus.

We then tested if mutations in a few regulators of cutin and VLCFA deposition, down-regulated in *tt16* or *tt1* backgrounds, displayed defects in IAB deposition. *myb30* mutant seeds appeared undistinguishable from wild type seeds after auramine O staining (Additional file 1: Figure S9A) and overexpression of *MYB30* in *tt16* seeds did not complement the *tt16* IAB phenotype (Additional file 1: Figure S9B and C). Likewise, auramine O staining of *wri1;wri3;wri4* seeds did not show IAB defects (Additional file 1: Figure S9D and E). Consistent with the absence of wax-specific compounds in our seed analyses, the overexpression of *DEWAX*, which represses wax biosynthesis in stems and leaves [29, 33], did not affect IAB deposition (Additional file 1: Figure S9F and G) and *tt16;dewax* double mutant did not complement the *tt16* IAB phenotype (Additional file 1: Figure S9H), when analyzed by auramine O staining.

Altogether, these results show that TT16 and TT1 regulate the expression of a number of genes involved in cutin deposition, which might be responsible for *tt16* and *tt1* IAB defects.

### *TT1* is specifically expressed in sporophytic maternal tissues

**Fig. 6:**
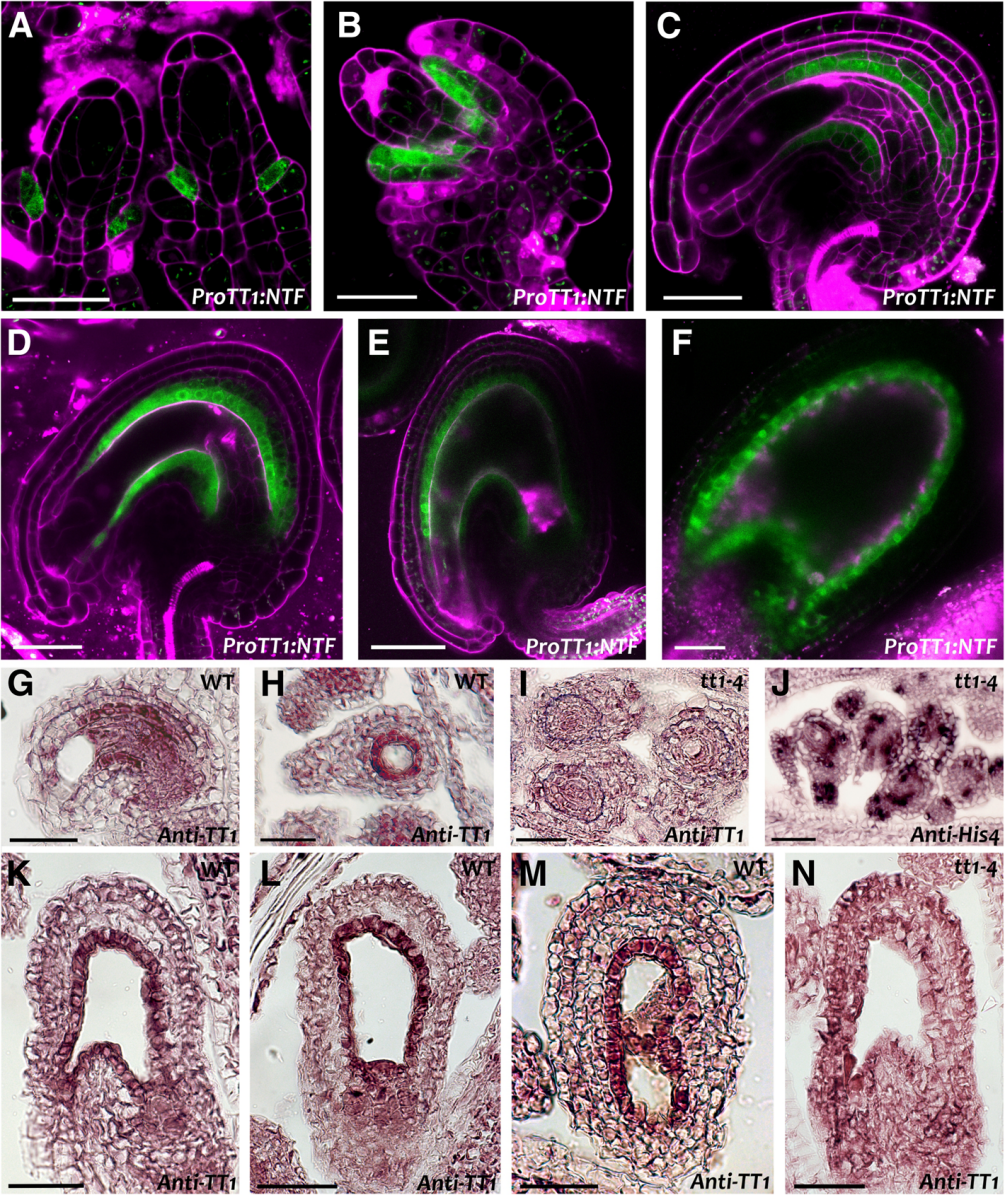
*TT1* expression in the integuments. **a** to **f** Fluorescence images of *ProTT1:NTF* ovules (**a** to **c**) and seeds (**d** to **f**). Green, NTF fluorescence; purple, **a** to **e** propidium iodide, **f** auto-fluorescence. Ecotype Col. **g** to **n** RNA in situ hybridization analyses of wild type and *tt1–4* mutant ovules and seeds with *TT1* and *HIS4* antisense probes. (**g**) and (**h**) Wild type ovules at 0 DAF. (**i**) and (**j**) *tt1–4* ovules at 0 DAF. (**k**) to (**m**) Wild type seeds at 1 DAF. (**n**) *tt1–4* seed at 1 DAF. Ecotype Ws. Scale bars: (**a**) and (**b**) 20 μm, (**c**) and (**d**) 30 μm, (**e**) to (**n**) 50 μm

We have previously shown *TT16* specific expression in the endothelium, the ii1’ cell layer and the nucellus using promoter-gene-*uidA* and *GFP* marker lines, as well as RNA in situ hybridization analyses [11, 63]. To confirm that *TT1* is also specifically expressed in seed maternal tissues, we transcriptionally fused the 1.1 kb *TT1* promoter region to the *NTF* tag sequence, coding for a GFP-containing chimeric protein that binds to the nuclear membrane (*ProTT1:NTF*) (Fig. 6) [15]. *ProTT1:NTF* seeds displayed strong fluorescence in the endothelium from the first stages of ovule development until globular embryo stage (Fig. 6a to f), as previously shown with a *ProTT1:uidA* marker line [53]. In addition, we observed a fainter signal in the other inner integument cell layers of mature ovules (Fig. 6d).

We further studied *TT1* expression pattern in ovules and seeds by RNA in situ hybridization analyses, using a probe specific to the 5′ region of *TT1* cDNA and *tt1–4* mutant as negative control (Fig. 6g to n). Before fertilization, we detected *TT1* mRNA in wild type endothelium, ii1’ cell layer and nucellus (Fig. 6g and h), but not in the *tt1–4* mutant (Fig. 6i). As a positive control, we hybridized *tt1–4* ovule sections with a *HISTONE4* (*HIS4*) antisense probe and observed its characteristic patchy expression pattern in actively dividing cells (Fig. 6j). After fertilization, *TT1* expression was restricted to the endothelium (Fig. 6k to m) and absent in the negative control (Fig. 6n). Importantly, we did not detect any signal in the wild-type central cell and endosperm. Taken together, these data indicate that *TT16* and *TT1* expression in ovules and seeds is specific to sporophytic maternal tissues.

### *TT16* cell and non-cell autonomous effects

**Fig. 7:**
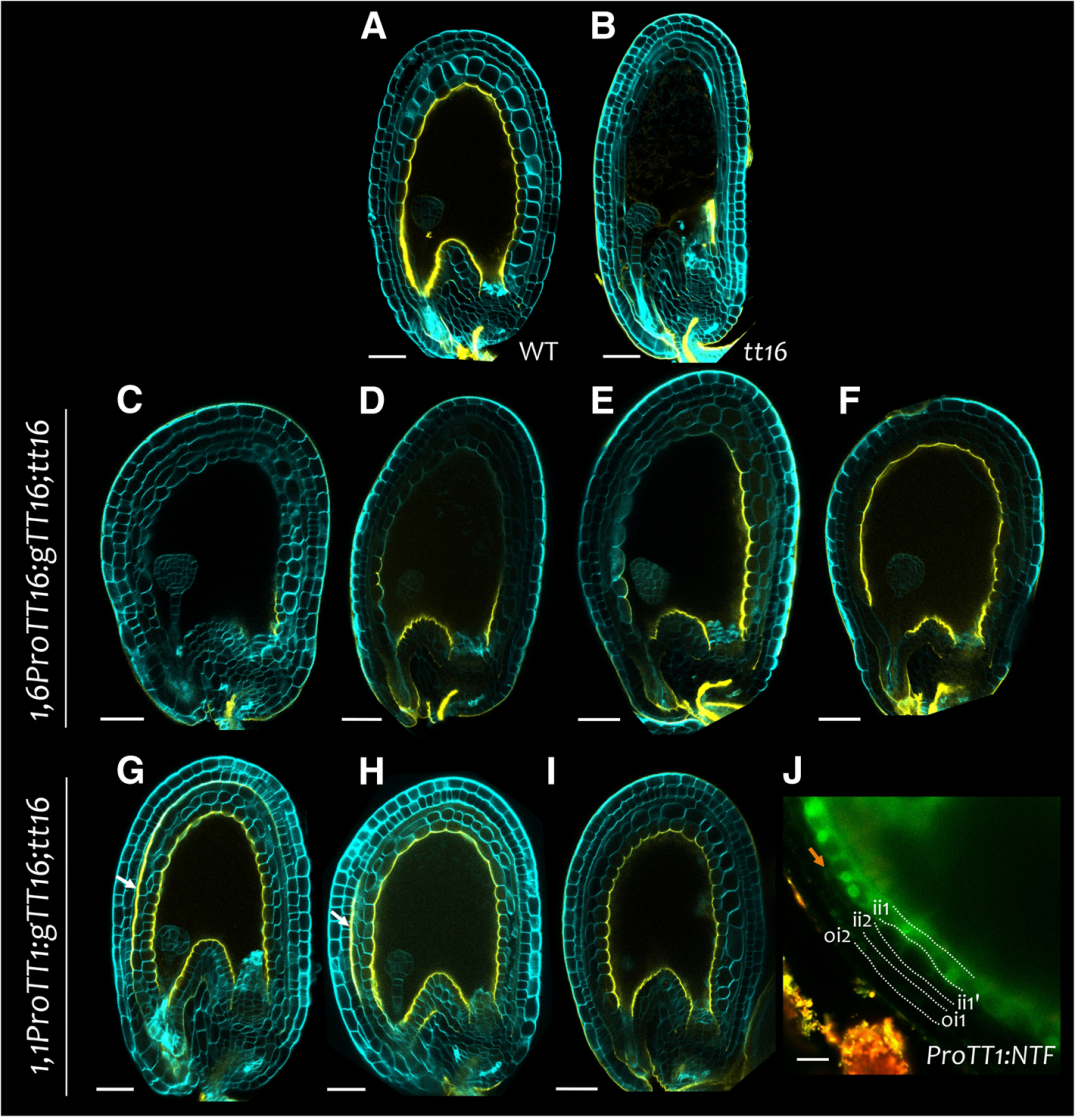
TT16 cell and non-cell autonomous effect on IAB deposition. **a** to **i** Fluorescence images of longitudinal sections of globular embryo stage seeds stained with auramine O (yellow) and counterstained with calcofluor (cyan). (**a**) Wild type. Ecotype Col. (**b**) *tt16*. Ecotype Col. **c** to **f** Representative sections of *1,6ProTT16:gTT16;tt16* seeds. Ecotype Ws. **g** to **i** Representative sections of *ProTT1:gTT16;tt16* seeds. Ecotype Ws. **j** Fluorescence image of a *ProTT1:NTF* seed coat. The orange arrow indicates a nucleus expressing GFP in the inner integument 2 (ii2) cell layer. Ecotype Col. oi, outer integument. Scale bars: (**a**) to (**i**) 50 μm, (**j**) 20 μm

We have previously shown that *TT16* expression in the ii, under the control of the *TT1* 1.1 kb promoter region, complemented *tt16* phenotypes in the nucellus and the ii1’ cell layer [11, 63]*.* By contrast, we observed partial complementation of the ii1’ cell layer phenotype when *TT16* was expressed in the nucellus and the first two or three most proximal cells of the endothelium under the control of its own 1.6 kb promoter region [63]. These data indicate that TT16 works both cell and non-cell autonomously. To assess the mechanism of action of TT16 in IAB deposition, we analyzed *tt16* lines carrying either a *1.6ProTT16:gTT16* or a *ProTT1:gTT16* construct (Fig. 7). Three independent *1.6ProTT16:gTT16;tt16* lines displayed heterogeneous complementation of the *tt16* IAB defects, ranging from faint auramine O staining in the proximal region to a wild type-looking phenotype (Fig. 7c to f). By contrast, three independent *ProTT1:gTT16;tt16* lines produced seeds fully complemented in IAB deposition (Fig. 7g to i). Two of the latter lines contained a fraction of seeds displaying strong auramine O staining in some parts of the MAB, a phenotype never observed in wild type and mutant seeds (Fig. 7g and h). This phenotype might be due to *TT16* ectopic expression in the ii2 under the control of the *TT1* promoter (Fig. 7j). Altogether, these data demonstrate that TT16 can regulate IAB deposition non-cell autonomously along the proximal-distal axis and accentuate MAB deposition in a cell-autonomous fashion.

### Sporophytic maternal action of *TT16* and *TT1*

**Fig. 8:**
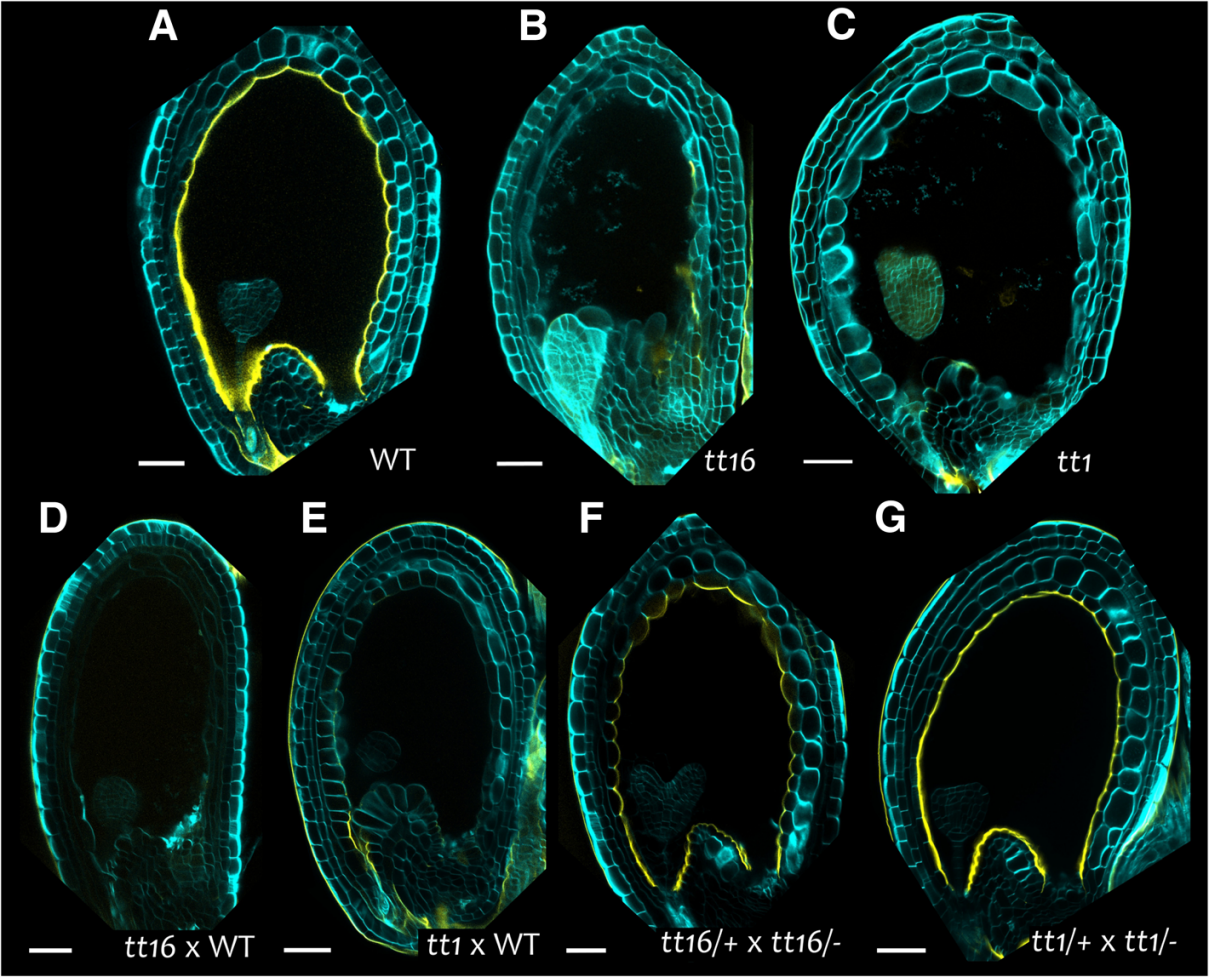
The IAB maternal origin. **a** to **g** Fluorescence images of longitudinal sections of globular embryo stage seeds stained with auramine O (yellow) and counterstained with calcofluor (cyan). (**a**) Wild type seed at 6 DAF. (**b**) *tt16* seed at 6 DAF. (**c**) *tt1* seed at 6 DAF. (**d**) Progeny seed at 6 DAP from a *tt16/−* x wild type (WT) cross. (**e**) Progeny seed at 6 DAP from a *tt1/−* x WT cross. (**f**) Progeny seed at 6 DAP from a *tt16/+* x *tt16/−* cross. (**g**) Progeny seed at 6 DAP from a *tt1/+* x *tt1/−* cross. Ecotype Col. Scale bars: 50 μm

*TT16* and *TT1* expression and function in integument development suggest their sporophytic maternal action on IAB deposition. To gain further insights, we crossed *tt16* and *tt1* plants with wild type pollen and analyzed their progeny seeds by auramine O staining (Fig. 8). All seeds analyzed (which carried *tt16/−* or *tt1/−* mutant maternal tissues and *tt16/+* or *tt1/+* heterozygous fertilization products) were undistinguishable from their respective *tt16/−* and *tt1/−* mutant seeds (Fig. 8d and e, compared to Fig. 8b and c, respectively). By contrast, seeds obtained by crossing *tt16/+* heterozygous plants with *tt16/−* mutant pollen (which carried *tt16/+* maternal tissues, and either *tt16/+* or *tt16/−* fertilization products) were undistinguishable from wild type seeds when stained with auramine O (Fig. 8f, compared to Fig. 8a). Likewise, seeds derived from the cross of *tt1/+* plants with *tt1/−* pollen displayed a wild type IAB (Fig. 8g, compared to Fig. 8a). These observations demonstrate that proper seed IAB deposition requires *TT16* and *TT1* expression in sporophytic maternal tissues.

### The fertilization of the central cell is sufficient for de novo IAB deposition

**Fig. 9:**
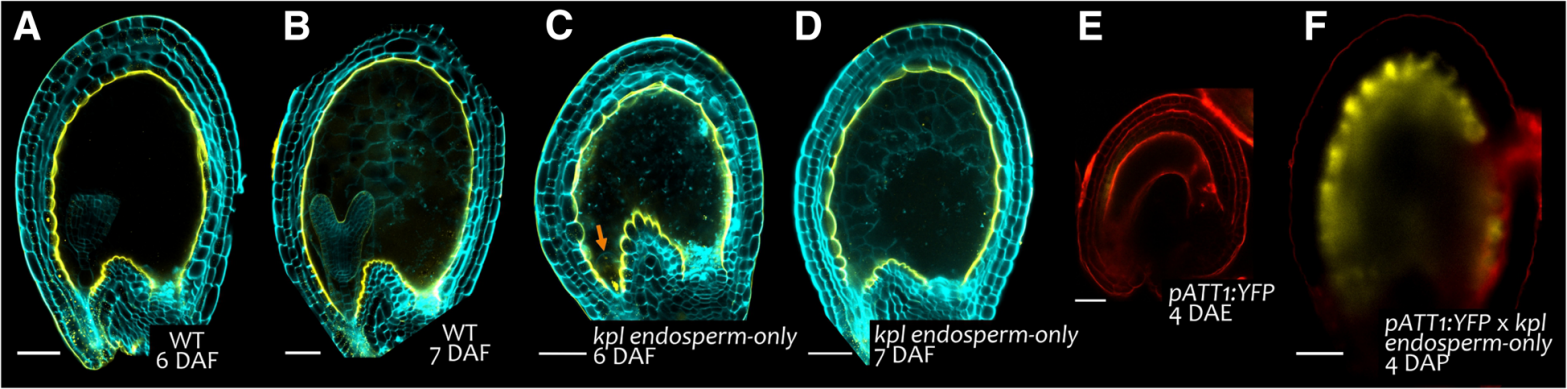
De novo cutin deposition in the IAB is triggered by central cell fertilization. **a** to **d** Fluorescence images of longitudinal sections of seeds and emasculated ovules, stained with auramine O (yellow) and counterstained with calcofluor (cyan). (**a**) and (**b**) Wild type seeds. Ecotype Ws. (**c**) and (**d**) *kpl* endosperm-only seeds. The orange arrow indicates the unfertilized, expanded egg-cell. Ecotype Ws. **e** Fluorescence image of a *ProATT1:YFP* ovule at 4DAE. Yellow, YFP; red, propidium iodide. Ecotype Col. **f** Fluorescence image of a progeny endosperm-only seed at 4DAP from a *ProATT1:YFP* (col) x *kpl* (Ws) cross*.* Yellow, YFP; red, propidium iodide. Scale bars: (**a**) to (**d**) and (**f**) 50 μm, (**e**) 30 μm

It has been shown that the fertilization of the central cell triggers endothelium growth and differentiation, with no apparent contribution of the embryo [52]. To test whether de novo IAB deposition, as part of the development program of the endothelium, is also triggered by endosperm development, we analyzed *kokopelli* (*kpl*) mutant seeds (Fig. 9). The *kpl* mutant is affected in pollen spermatic cell development and characterized by a percentage of random single-fertilization events, thus producing seeds carrying either the endosperm or the embryo [51]. Whereas *kpl* embryo-only seeds fail to develop any further, endosperm-only seeds undergo cell expansion and produce PAs [52]. Similarly, the IAB of *kpl* endosperm-only seeds was undistinguishable from that of wild type seeds after auramine O staining, suggesting that central cell fertilization is sufficient for correct IAB deposition (Fig. 9a to d). To test whether *kpl* endosperm-only seeds undergo de novo cutin deposition in the IAB, we fertilized *ProATT1:YFP* transgenic plants with *kpl* pollen and analyzed the progeny. A strong *ATT1* promoter activity was detected in the endothelium of all analyzed *ProATT1:YFP;kpl* endosperm-only seeds at 4 DAP (Fig. 9f), whereas unfertilized ovules at 4 DAE exhibited the same faint fluorescence near the micropyle as ovules at anthesis (Fig. 9e). These data indicate that the fertilization of the central cell is sufficient to trigger de novo cutin deposition in the IAB.

An endosperm signal is thought to relieve the repressive mechanism mediated by FERTILIZATION INDEPENDENT SEED (FIS) Polycomb group (PcG) proteins, which prevent the fertilization-independent development of ovules [52]. Among FIS PcG proteins, FERTILIZATION INDEPENDENT ENDOSPERM (FIE) and MULTICOPY SUPPRESSOR OF IRA1 (MSI1) act sporophytically to repress integument differentiation in ovules [52]. FIE and MSI1 are haploinsufficient and a fraction of *fie/+* and *msi1/+* ovules develop into large autonomous seeds without any fertilization. The endothelium of such autonomously-developed seeds undergoes cell expansion and produces PAs [52]. To test whether FIE and MSI also repress de novo cutin deposition in the IAB, we analyzed *fie/+* and *msi1/+* autonomously-developed seeds at 6 DAE. After staining with auramine O, the IAB of *fie/+* and *msi1/+* large autonomously-developed seeds appeared undistinguishable from that of wild type fertilized seeds (Additional file 1: Figure S10A and B). Moreover, *tt16;fie/+*, *tt1;fie/+*and *tt1;msi/+* large autonomously-developed seeds exhibited the same auramine O staining pattern as their respective *tt16* and *tt1* single mutants, thus indicating that *TT16* and *TT1* are epistatic to *FIE* and *MSI* (Additional file 1: Figure S10C to 10E). Altogether, these data indicate that IAB deposition and PA biosynthesis are regulated by the same signaling pathway.

### Impaired IAB deposition affects embryo development in *tt16* seeds

**Fig. 10:**
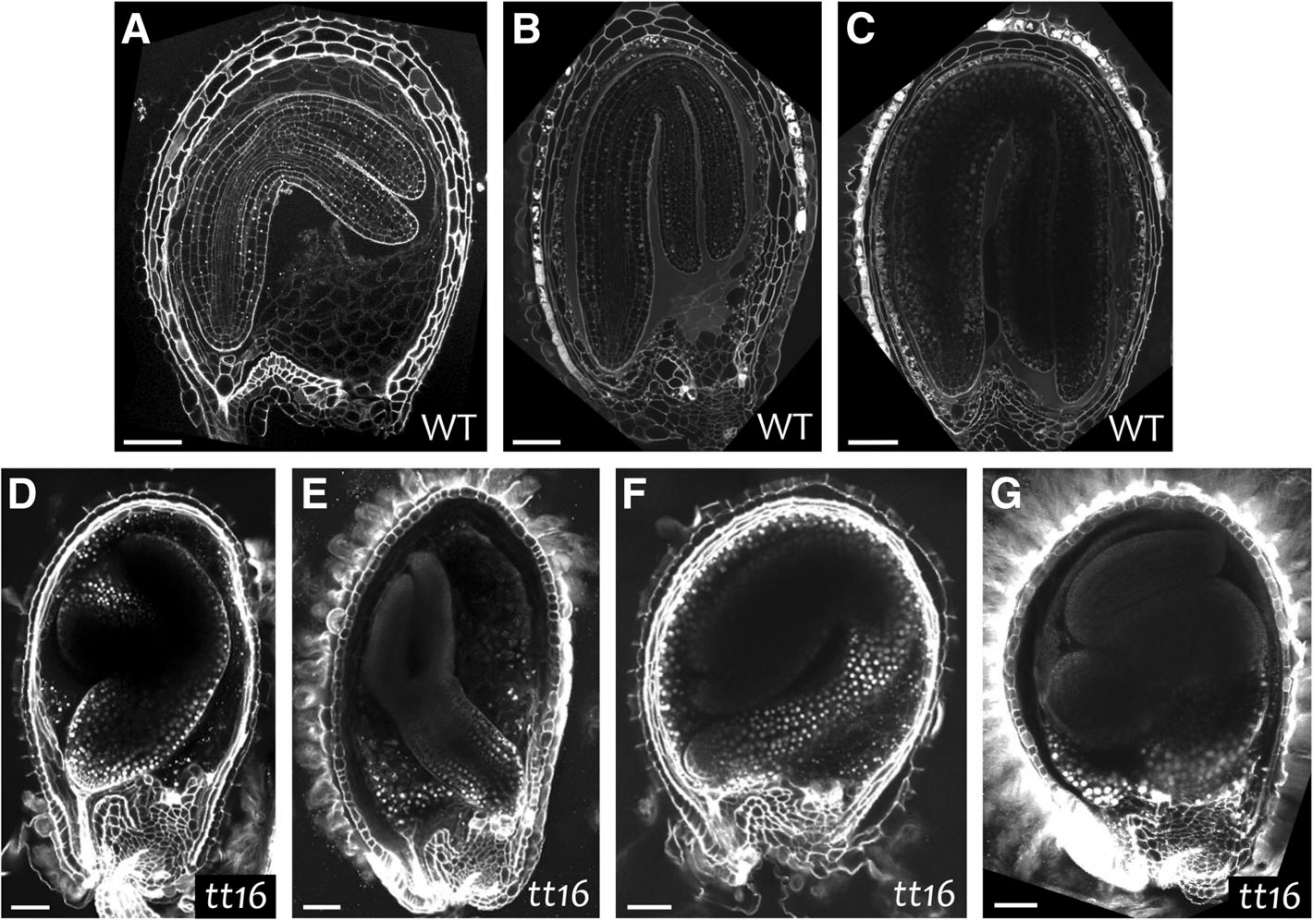
Impaired IAB deposition in *tt16* seeds affects embryo development. **a** to **g** Fluorescence images of wild type and *tt16* seeds longitudinal sections imaged using the mPS-PI technique.WT, wild type. Ecotype Ws. Scale bars: 50 μm

It was previously shown that defective embryo cuticle formation can lead to the adhesion of the embryo to the adjacent endosperm cells and impair its development [25, 62, 64]. To test whether lack of IAB deposition can similarly affect the development of the fertilization products, we analyzed wild type and *tt16* seeds at late torpedo and early bent cotyledon embryo stages using the modified pseudo-Schiff propidium iodide (mPS-PI) imaging technique (Fig. 10) [63]. In all analyzed wild type seeds, embryos developed alongside the seed coat from the micropyle toward the chalaza (Fig. 10a to c). By contrast, a 21% of *tt16* seeds (*n* = 100) displayed embryos apparently attached to the inner cell wall of the seed coat and incorrectly twisted, a phenotype reminiscent of cuticle-less embryo mutants (Fig. 10d to g) [25, 62, 64]. Furthermore, in some *tt16* seeds, the developing radicle was displaced from the micropylar to the chalazal region (Fig. 10e). These phenotypes might be due to abnormal physical tensions resulting from both embryo growth and cotyledon adhesion to the endothelium, thus suggesting that lack of IAB deposition can impair embryo development.

## Discussion

In angiosperms, seeds are composed of three genetically distinct compartments: embryo, endosperm and maternal tissues. Mounting evidences indicate the presence of an intricate signaling network underlying the coordinated development of such tissues. Nonetheless, little is known on the physical interfaces separating different seed compartments. Here we characterized the deposition of apoplastic lipid barriers in ovule and seed maternal tissues. Moreover, we demonstrated the sporophytic maternal function of two transcription factors, TT1 and TT16, in regulating the deposition of the apoplastic barrier (IAB) that separates maternal tissues from fertilization products.

### Deposition of apoplastic lipid barriers in the seed coat

We identified apoplastic lipid barriers surrounding ovule and seed maternal tissues by TEM and auramine O staining (Fig. 1). The apoplastic barrier (IAB) that separates the inner integument from the nucellus in ovules and from the endosperm in seeds was detectable at all developmental stages analyzed (Fig. 1). Nevertheless, its thickness and auramine O fluorescence appear to gradually diminish during development, especially in the seed curving zone. This might be due to the stretching of the IAB as a result of endothelium cell expansion following fertilization. By contrast, the apoplastic barrier laying in between oi and ii (MAB) exhibited large gaps in mature ovules (Additional file 1: Figure S4) and almost completely disappeared in seeds, in line with what previously reported [13].

Furthermore, we investigated IAB composition by GC-MS, comparing wild type to mutants affected in IAB deposition. We did not detected VLCFA derivatives, typical of waxes, in our analysis (Fig. 2a and Fig. 4). An explanation could be that only a low amount of wax, below our threshold detection, is deposited. Alternatively, wax deposition might occur only after torpedo embryo stage of seed development. Wax might also be a unique feature of cuticles that are in direct contact with the outside environment, and be unnecessary or detrimental inside the seed. GC-MS analysis revealed instead a progressive enrichment in C18:2 DCA during seed development (Fig. 2a). In addition, the exclusive sporophytic maternal effect of TT16 and TT1 (Fig. 6 to Fig. 8) and the drastic drop in C18:2 DCA content in *tt16* and *tt1* mutant seeds at 4 DAF (Fig. 4) suggest that C18:2 DCA is mostly specific to maternal tissues, at least at globular embryo stage of seed development.

Finally, *ATT1*, a gene encoding a key enzyme in the biosynthetis of cutin and critical for C18:2 DCA deposition [42], exhibited an endothelium-specific expression in seeds (Fig. 2c to e). This result suggests that C18:2 DCA is specifically produced in the endothelium in the early steps of seed development. Besides, the IAB structure, as observed by TEM, resembled that of many cutin layers studied to date (Fig. 1k to p), exception made for the absence of an epicuticular wax layer, and differed from that of usual suberin layers [26, 44]. Overall, these findings show that the seed IAB is an internal apoplastic barrier made of cutin, and with no or little intracuticular waxes.

### De novo IAB deposition following fertilization

GC-MS analyses showed a de novo C18:2 DCA accumulation following fertilization (Fig. 2a). Furthermore, RT-qPCR analyses revealed an important rise in the expression of genes involved in cutin deposition, such as *ATT1*, *BDG*, *GPAT4*, *LACS2*, *WBC11* and *WRI4*, after fertilization (Fig. 2b). The expression of *ATT1*, which encodes for an enzyme involved in cutin biosynthesis, progressively increased by 20 folds from 0 DAF to 4 DAF and its promoter showed a much stronger endothelium activity after fertilization (Fig. 2c to e), which was never observed in ovules at 4 DAE (Fig. 9e). By contrast, the expression of the *WRI4* transcription factor gene picked at 2 DAF, thus suggesting that it plays an early role in the transcriptional regulation of cutin biosynthesis. Finally, the fertilization-dependent activation of such cutin-related genes is further demonstrated by previous transcriptomic data comparing ovules of emasculated flowers to fertilized seeds [22]. Altogether, these data demonstrate that fertilization triggers de novo cutin deposition in the IAB.

**Fig. 11:**
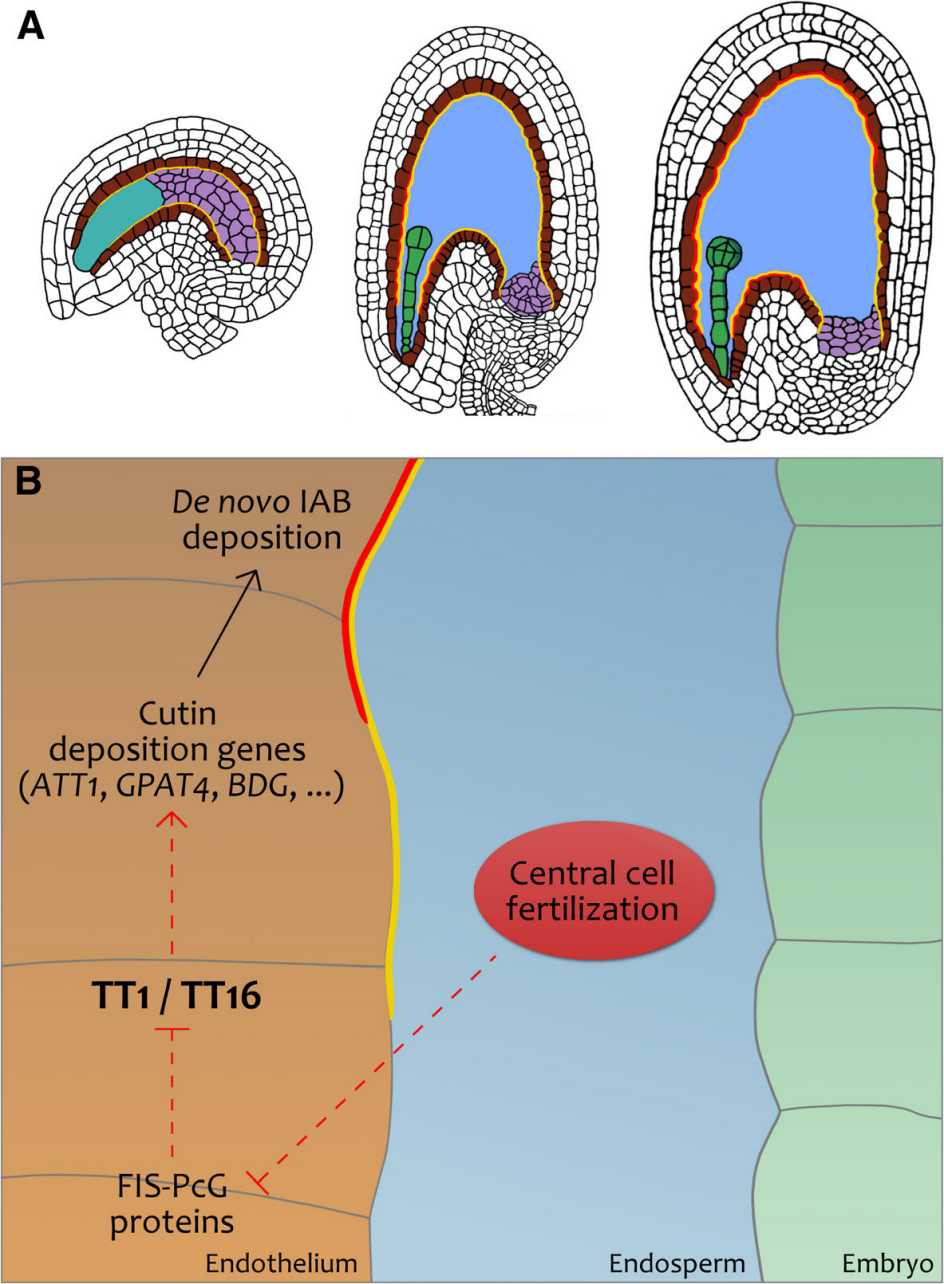
A model for IAB deposition. **a** A schematic of IAB deposition from ovule to seed. Purple, nucellus; turquoise, female gametophyte; brown, endothelium; blue, endosperm; green, embryo. The yellow line represents the IAB deposited during ovule development, whereas the red line marks the newly deposited IAB after fertilization **b** Genetic pathway regulating IAB deposition. Black and red arrows indicate functional and molecular relationships, respectively. Dashed arrows indicate unknown regulatory mechanisms.

It has been previously demonstrated that central cell fertilization drives seed coat growth and differentiation [22, 23]. In line with this study, *kpl* endosperm-only seeds displayed the same auramine O staining and *ATT1* expression pattern as wild type seeds (Fig. 9a, b and d). These results suggest that a signal originating from the fertilization of the central cell is sufficient to trigger de novo cutin deposition in the IAB (Fig. 11). Nevertheless, a role of the embryo in the IAB composition after fertilization cannot be excluded.

### TT16 and TT1 promote IAB deposition

TT16 and TT1 are regulators of integument development and promote PA accumulation in the endothelium [11, 45, 53]. We showed that TT16 and, to a lesser extent, TT1 promote IAB deposition already before fertilization. After fertilization, we observed differences in IAB deposition between *tt16* and *tt1* mutations along the seed proximal-distal axis. Importantly, both mutations showed a strong phenotype along the seed curving zone. This might be due to the stretching of the IAB in the seed region that undergoes the most dramatic cell elongation, concomitantly with a reduced or absent de novo IAB deposition in both mutant seeds. Furthermore, we observed a strong reduction in C18:2 DCA content in *tt16* and *tt1* seeds, compared to wild type. In line with these results, the expression of several genes involved in cutin biosynthesis was significantly reduced in both mutant lines. Overall, these data indicate that TT16 and TT1 positively regulate the deposition of cutin in the IAB (Fig. 11).

### IAB maternal origin

It has been previously suggested that the IAB of mature seeds is deposited by the endosperm [14]. However, a recent study challenged such interpretation and proposed its origin from maternal tissues [38]. Microarray analyses performed by Belmonte and coworkers support the latter conclusion [6] as they show that a large set of genes involved in cuticle deposition are more expressed in the seed coat than in the endosperm [6, 30]. We also gathered data in favor of the IAB maternal origin. First, we detected a IAB on ovule integuments and nucellus till fertilization. Besides, *ATT1*, a key gene in cutin biosynthesis, was specifically expressed in the endothelium. Finally, *TT16* and *TT1* were both expressed in maternal tissues and acted maternally to induce IAB deposition (Fig. 11). Although some contribution from the endosperm cannot be excluded, these results demonstrate that the IAB is produced by the endothelium to a wide extent.

### Transcriptional regulation of IAB deposition

MYB30 and members of the WRINKLED transcription factor family act upstream of cutin-related genes [50, 56] and are strongly expressed in the Arabidopsis seed coat [6], thus being putative candidate regulators of IAB deposition. Nevertheless, IAB deposition was not affected by *myb30* or *wri1;wri3;wri4* mutations. On the other hand, *WRI3* and *WRI4* expression was depleted in both *tt16* and *tt1* seeds, as did the expression of *MYB30* in *tt16.* In addition, we detected *ProMYB30:gMYB30-uidA* activity in the endothelium of wild type but not *tt16* seeds. *tt16* IAB phenotype could not be complemented by *MYB30* overexpression, possibly due to the previously reported tight post-translational regulation of MYB30 [27, 40] or to genetic redundancy. Taken together, these results strongly suggest that IAB deposition is controlled by a multiple transcriptional regulators that act downstream of TT16 and TT1 (Fig. 11).

### IAB physiological function

De Giorgi and coworkers demonstrated that IAB deposition in mature seeds has an impact on seed permeability, water uptake, and testa rupture following imbibition [14]. Their model proposes that the IAB acts as a waterproof barrier, isolating the endosperm and the embryo from water and dioxygen. This would prevent production of reactive oxygen species, thus delaying dormancy breakdown and seed aging [14]. In line with this interpretation, we never detected gaps along the IAB, neither with auramine O staining nor in TEM. Nonetheless, further analyses on IAB permeability have to be conducted to better understand its role as diffusion barrier to nutrients, hormones and other solutes speculated to be exchanged between seed coat and nucellus or endosperm. Interestingly, we observed lack of auramine O staining in the distal micropylar region of wild type ovules and seeds (Fig. 1), as previously described in mature seeds [38]. The absence of IAB in the distal micropylar region suggests that this region might be adapted to signal exchange. One might indeed assume that the transfer of specific fertilization signals from the newly formed endosperm to the integuments occurs in this area. In line with this hypothesis, *ATT1* and *MYB30* promoters displayed activity patterns similar to that of *BANYULS*, a key gene in PA biosynthesis, starting in the distal region and then spreading to more proximal regions of the endothelium [16]. Similarly, the distal surface of the seed nucellus and the pigment strand are not covered by a cuticle layer, thus offering another possible entrance to fertilization signals.

One of the crucial functions of cuticles is to prevent the fusion of tissues developing side by side [30]. A fraction of *tt16* seeds displayed twisted embryos with the cotyledons apparently attached to the seed coat. Such a phenotype is reminiscent of mutants defective in embryo cuticle formation [58, 64]. *TT16* expression pattern and sporophytic maternal action strongly suggest that the *tt16* embryo phenotype is due to defects in the seed coat and not zygotic developmental programs. We therefore hypothesize that an essential function of the IAB is to ensure correct sliding of the developing embryo along the seed coat. Similarly, the MAB might allow independent growth of ii and oi during early ovule development. Its later disappearance might instead facilitate the fusion of both integuments in a more compact seed coat.

## Conclusions

Proper seed formation requires spatial and temporal coordination of all seed tissues. The apoplastic space that separates different seed compartments is supposed to influence their communication. Here we unveil the nature, regulation, deposition pattern and function of a cutin apoplastic barrier that is at the interface between maternal tissues and fertilization products. This study opens new perspectives for the analysis of communication between seed tissues.

## Methods

### Plant material

*Arabidopsis thaliana* plants of ecotype Columbia (Col-0) or Wassilewskija (Ws-2) were used as wild type controls as appropriate. The *tt16–1* mutant was isolated in the Ws-2 accession and then backcrossed to the Col-0 accession more than three times [45, 63]. *tt1–3, ttg2–3, myb30–1, dewax*, *wri1–4;wri3–1;wri4–1*, *wri1–4;wri3–1;wri4–3, fie-12/+* and *msi1–1/+* mutants are in the Col-0 accession [1, 20, 29, 52, 56]. *kpl-1, tt16–2* and *tt1–4* mutants are in the Ws-2 accession [8, 45, 51]. The *ttg1–1* mutant is in the Ler accession [3]. *ProTT1:gTT16;tt16–1* and *1.6ProTT16:gTT16;tt16–1* lines are in the Ws-2 accession [63]. *ProTT1:NTF*, *DualPro35S:cMYB30;tt16–1*, *ProMYB30:gMYB30-uidA* and *ProMYB30:gMYB30-uidA;tt16–1* are in the Col-0 accession*. DEWAX-OX (Pro35S:cDEWAX) lines* are in the Col-0 accession [29]. Unless noted, *tt16* and *tt1* refer to *tt16–1* and *tt1–3* respectively, and Col was used as wild type. Arabidopsis seeds came from our seed collection at INRA Versailles. Collection of plant material complied with local and national guidelines.

Days after flowering were counted starting from the emergence of the pistil from closed flowers; 0 DAF equals stage 3-V of ovule development [54]. Emasculation was performed at 0 DAF.

### Cloning and construction

The *NTF* sequence was PCR amplified from *pMDC107-NTF* [39], *cMYB30* was PCR amplified from cDNA of Col-0 siliques, whereas *ProTT1* and *ProMYB30:gMYB30* were PCR amplified from Col-0 genomic DNA. All PCR products were amplified by high-fidelity Phusion DNA polymerase (Thermo Fisher Scientific). PCR amplifications of *NTF, cMYB30* and *ProMYB30:gMYB30* were performed using the gene-specific primers described in Additional file 1: Table S1, carrying the *attB1* (5′-GGGGACAAGTTTGTACAAAAAAGCAGGCT-3′) and *attB2* (5′-GGGGACCACTTTGTACAAGAAAGCTGGGTC-3′) Gateway recombination sites at the 5′-ends of the forward and reverse primers, respectively. The PCR products were recombined into the *pDONR207* vector (BP Gateway recombination) according to the manufacturer’s instructions (Thermo Fisher Scientific), and sequenced.

For the construction of the *pGWB2-ProTT1* destination vector, *Pro35S* was removed from *pGWB2* by digestion with HindIII High Fidelity and XbaI restriction enzymes (New England Biolabs). PCR amplification of *ProTT1* was performed using the gene-specific primers described in Additional file 1: Table S1, carrying 15 bp tails at their 5’ends matching digested *pGWB2* overhangs. The digested plasmid and the PCR product were spin-column purified after electrophoresis and extraction of the corresponding bands. *ProTT1* was cloned into digested *pGWB2* using InFusion HD Cloning Kit, according to the manufacturer’s instructions (Clontech). Cloning of *NTF* into *pGWB2-ProTT1*, *cMYB30* into *pMDC32*, and *ProMYB30:gMYB30* into *pGWB3* were performed by LR recombination according to the manufacturer’s instructions (Thermo Fisher Scientific).

### Transgenic plants

The *Agrobacterium tumefaciens* strain C58C1 was used to stably transform Arabidopsis plants using the floral dip method [10]. Transformants were selected on MS medium containing hygromycin (50 mg L^− 1^) and subsequently transferred to soil for further characterization.

### RNA extraction

Ovules and seeds used for total RNA extraction were frozen in liquid nitrogen immediately after harvest and stored at − 80 °C prior to extraction. Four independent biological samples were used for each analysis. Each replicate comprised the content in ovules/seeds of 10 to 15 pistil/siliques. Total RNA was extracted using the RNeasy Mini kit (Qiagen), including RNase-Free DNase Set (Qiagen) treatment during washing, according to the manufacturer’s instructions, and subsequently stored at − 80 °C.

### Expression analysis

The Superscript Reverse Transcriptase II kit (Invitrogen) was used to generate cDNA from 1 μg of total RNA. Each cDNA sample was diluted 1:125 in water. Quantitative PCRs were performed with the SYBR Green kit (Bio-Rad) on a Bio-Rad CFX real-time PCR machine. For each reaction, 4.4 μL of diluted cDNA were added to 5 μl of SYBR Green and to 0.3 μl of each primer (10 μM) (Additional file 1: Table S1). Expression levels were first normalized by the geometrical mean of the expression levels of the 4 reference genes chosen (*GAPDH*, *AT4G12590*, *AT4G02080* and *AT3G25800*; [18]), and subsequently normalized by the expression level of the adequate control. Means and standard deviations were calculated from the values obtained for the 4 independent biological samples.

### Biochemical analysis

Pistils were dissected and ovules and seeds were collected and placed in glass vials on ice. Samples were subsequently stored at − 80 °C for several days and lyophilized. Analysis of total seed lipid content was performed without delipidation. Following steps were performed as previously described [19]. Two independent biological samples were performed (three for wild type at 4 DAF), each one comprising the seed content of 10 to 15 pistils/siliques. Concerning the additional GC-MS analysis in Additional file 1: Figure S7, samples were kept at − 80 °C without lyophilization and 6 independent biological samples were used for both wild type and *tt16* seeds.

### Sample preparation for microscopy

Prior to microscopy analyses, siliques were carefully dissected and septums (containing seeds) were harvested.

For staining with auramine O (Sigma Aldrich) and calcofluor M2R white (fluorescent brightener 28; Sigma Aldrich), harvested septums were first immersed in a NaOH (0.2 M), SDS (1%) solution at 37 °C for 3 h for seeds and 2 h for ovules. Samples were washed three times in water, and then transferred to a fresh bleach solution (2%) for 10 min to remove precipitated tannins in seeds (this step was skipped for ovules). Samples were washed at least five times to remove traces of bleach (which interferes with auramine O staining). Finally, they were immersed in a staining solution containing auramine O (10 μg mL^− 1^) and calcofluor M2R white (10 μg mL^− 1^) at 4 °C overnight and mounted in water before analysis. Stock solutions (100 μg mL^− 1^) of Auramine O and Calcofluor M2R White were stored at − 20 °C for up to 6 months.

Seeds of *ProTT1:NTF* and *ProATT1:YFP* lines were analyzed 1 h after mounting in a Propidium iodide (100 μg mL^− 1^), sucrose (7%) solution, as previously described [22]. For analysis of *ProMYB30:gMYB30-uidA* lines, seeds were immersed in solution n°1 (Additional file 1: Table S2). Samples were subsequently mounted on slides in a chloral hydrate 8 M, glycerol 33% solution. mPS-PI samples were prepared as previously described [63].

For transmission electron microscopy (TEM), septums with seeds were immersed immediately after harvest in a fresh fixative solution (solution n°2, Additional file 1: Table S2). Fixation was performed for 4 h at room temperature and 1 week at 4 °C. Samples were subsequently contrasted with Oolong Tea Extract (OTE) (Delta Microscopies – France) 0.5% in cacodylate buffer 0.1 M pH 7.4 for 1 h, post-fixed with 1% osmium tetroxide containing 1.5% potassium cyanoferrate for 2 h, gradually dehydrated in ethanol series (10 to 90%, 1 h for each bath), and dehydrated twice for 1 h in ethanol 100%. Samples were then gradually treated with mixtures of ethanol-epon (ratios of 1:2, 1:1 and 2:1, for 2 h time) and finally transferred to pure epon (Delta Microscopie) overnight under vacuum. For embedding, ovules/seeds were spread on silanized glass slides. One drop of epon was added before overlaying with a second glass slide.

After polymerisation (48 h at 56 °C), the epon layer was removed from the slide. Selected ovules and seeds were cut out and stuck on the top of Beem capsules (EMS) pre-filled with epon. Semi-thin sections (500 nm) were collected and colored with azure II/methylene blue to check tissue integrity before thin sections. Thin sections (70 nm) were collected onto either 125/200 mesh copper grids or slot grids and counter-stained with lead citrate.

### RNA in situ hybridizations

*TT1* and *HIS4* antisense probes were PCR amplified by high-fidelity Phusion DNA polymerase (Thermo Fisher Scientific) using forward and reverse primers listed in Additional file 1: Table S1. Probe purification was performed as previously described [61].

After harvest, septums were immediately immersed in a fresh fixative solution (solution n°3, Additional file 1: Table S2), vacuumed 4 times, immersed in a new fresh fixative solution (solution n°3, Additional file 1: Table S2) and incubated overnight at 4 °C. Samples were then gradually dehydrated in ethanol series (10, 30, 50, 70, 96%) and incubated overnight in ethanol 96% with eosin (Sigma, 0.1%). They were immersed three times in ethanol 100% for 2 h, subsequently treated with a ethanol/histoclear (National Diagnostics) series of 2:1, 1:1 and 1:2 (1 h each time), transferred to three consecutive baths of pure histoclear (20 min each), then to a mix of histoclear and paraffin 1:1 for 1 h at 59 °C, followed by pure paraffin overnight at 59 °C. Samples were transferred to a second paraffin medium for 3 h at 59 °C, to a third one at 59 °C overnight, finally included in molds in fresh paraffin and stored at 4 °C before sectioning. 8 μm sections were performed using a carbon blade on a Leica RM2055 microtome and subsequently placed on slides, previously covered with water drops, at 37 °C overnight. After water evaporation, slides were stored at 4 °C.

Slides were immersed into two baths of pure histoclear (10 min and 15 min respectively), then in 100% ethanol (1 min), subsequently rehydrated in decreasing ethanol series (100%, 96%, 85% + 0.42% Nacl, 70% + 0.85% NaCl, 50% + 0.85% Nacl, 30% + 0.85% Nacl) for 30 s each, and finally immersed in 0.85% Nacl for 2 min. Samples were then consecutively transferred to a PBS solution (2 min), to a proteinase K solution (solution n°4, Additional file 1: Table S2, 10 min at 37 °C), to a glycine 0.2% solution in PBS (2 min), to an acetic anhydride/triethanolamine-HCL solution (solution n°5, Additional file 1: Table S2), and eventually to a new PBS solution (2 min). Drops of prehybrization buffer (solution n°6, Additional file 1: Table S2) were deposited on slides and incubation was performed at hybridization temperature for 1.5 h. The RNA probes were added to the hybridization buffer (solution n°7, Additional file 1: Table S2) and, following denaturation (2 min at 80 °C), drops of this solution were pipetted on slides. A second slide was placed onto the first one, and samples were incubated overnight at hybridization temperature in a damp box containing a formamide 50%/10X SSC solution. Slides were then consecutively washed in 0.1X SSC/0.5% SDS (30 min, 56 °C), in 2X SSC/50% formamide (2 h, 56 °C), in NTE (5 min, 56 °C), in a RNAse solution (solution n°8, Additional file 1: Table S2, 30 min, 37 °C), in NTE again (5 min, 56 °C), in formamide 50%/2X SSC (1 h, 56 °C), in 0.1X SSC (2 min, 56 °C) and finally in PBS overnight at room temperature. Immunological detection was performed by transferring slides to solution n°9 (Additional file 1: Table S2, 1 h, room temperature), then to solution n°10 (Additional file 1: Table S2, 1 h, room temperature), and finally by pipetting drops of digoxygenin-targetting antibody solution on slides (solution n°11, Additional file 1: Table S2, incubation 1 h, room temperature). Slides were then consecutively washed at room temperature in solution n°10 (Additional file 1: Table S2, 2 times for 20 min), in a Tris pH 7.5 (100 mM)/NaCl (150 mM) solution (15 min), and in solution n°12 (Additional file 1: Table S2, 15 min). Staining reaction was performed by dipping slides in solution n°13 (Additional file 1: Table S2) for 24 h, and subsequently stopped by incubation in TE at pH 7.5 for 20 min. Slides were rinsed with permuted water, mounted in Citifluor AF1 and stored at 4 °C.

### Microscopy

mPS-PI stained samples were analyzed with a Leica TCS-SP5 spectral confocal laser scanning microscope (Leica Microsystems, numerical aperture =1 and 60x objective). Samples stained with auramine O and calcofluor, as well as *ProTT1:NTF* and *ProATT1:YFP* lines were analyzed using a Leica TCS-SP8 spectral confocal laser scanning microscope, under sequential scanning (Leica Microsystems). Excitation and signal reception were set as previously described [9, 11, 23]. RNA in situ hybridization samples and *ProMYB30:gMYB30-uidA* lines were analyzed by DIC microscopy with an Axioplan 2 microscope (Zeiss). For TEM, samples were examined with Hitachi HT7700 electron microscope operated at 80 kV (Elexience – France), and images were acquired with a charge-coupled device camera (AMT).

For confocal microscopy, pictures showing mid-plane longitudinal sections of seeds were captured when possible. Otherwise, three dimensional z-stacks were acquired, and the mid-plane longitudinal sections were obtained with the Volume Viewer plugin of the Image J software (Rasband, W.S., ImageJ, U. S. National Institutes of Health, Bethesda, Maryland, USA, https://imagej.nih.gov/ij/, 1997–2018).

### Analysis of auramine O staining pattern in mutant seeds

Staining patterns in wild type, *tt16* and *tt1* were quantified with the Image J software. The length of both distal and proximal areas showing auramine O staining were measured and divided by the total length of the endothelium.

### Accession numbers

Sequence data from this article can be found in the GenBank/EMBL data libraries under the following accession numbers: *TT16* (AT5G23260), *TT1* (AT1G34790), *ATT1* (AT4G00360), *BDG* (AT1G64670), *DCR* (AT5G23940), *GPAT4* (AT1G01610), *LACS2* (AT1G49430), *FATB* (AT1G08510), *WBC11* (AT1G17840), *WRI1* (AT3G54320), *WRI3* (AT1G16060), *WRI4* (AT1G79700), *MYB30* (AT3G28910), *CER1* (AT1G02205), *CER5* (AT1G51500), *CER6* (AT1G68530), *KCS1* (AT1G01120), *MYB96* (AT5G62470), *DEWAX* (AT5G61590), *KPL* (AT5G63720), *FIE*(AT3G20740), *MSI1* (AT5G58230), *HIS4* (AT2G28740), *tt16–1* (INRA DXT32), *tt1–3* (SALK_026171), *tt1–4* (INRA DXL-6), *ttg2–3* (SALK_149938), *myb30–1* (SALK_122884), *ttg1–1* (N89), *wri1–4* (N508559), *wri3–1* (N656326), *wri4–1* (N518113), *wri4–3* (N546920), *dewax* (SALK_015182C), *kpl-1* (FST 184H02), *fie-12* (GK-362D08) and *msi1–1* (TAIR: 1510594109).

## Additional files

Additional file 1: **Figure S1.** Detection of the IAB without staining. **Figure S2.** Detection of the IAB in different Arabidopsis ecotypes. **Figure S3.** The ovule IAB is made of nucellus and endothelium apoplastic barriers. **Figure S4.** Detection of the MAB in ovules. **Figure S5.** Expression of a set of genes involved in VLCFA deposition in ovules and seeds. **Figure S6.** IAB deposition in *tt* mutant seeds. **Figure S7.** *tt16* seeds display an altered C18:2 DCA composition. **Figure S8.** *tt16* and *tt1* seeds exhibit altered expression of genes involved in VLCFA deposition. **Figure S9.** IAB deposition in mutant and over-expression lines of genes involved in cutin and wax deposition. **Figure S10.** IAB deposition is repressed by FIE and MSI1 FIS-PcG proteins. **Table S1.** Primers. **Table S2.** Solutions. **Table S3.** Student’s t test statistical analysis of GC-MS results. (PDF 1837 kb)

## Data Availability

The datasets used and/or analysed during the current study are available from the corresponding author on reasonable request.

## Abbreviations

ATT1: Aberrant induction of type three 1
BDG: Bodyguard
CER: Eceriferum
DAE: Days after emasculation
DAP: Days after pollination
DCA: Dicarboxylic fatty acids
DCR: Defective in cuticular ridges
DIC: Differential interference contrast
FIE: Fertilization independent endosperm
FIS: Fertilization independent seed
GPAT: Glycerol-3-phosphate sn-2-acyltransferase
HIS4: Histone4
IAB: Inner apoplastic barrier
ii1: Inner integument 1
KCS1: 3-Ketoacyl-coa synthase 1
KPL: Kokopelli
LACS2: Long-chain acyl-coa synthetase 2
MAB: Middle apoplastic barrier
mPS-PI: Modified pseudo-Schiff propidium iodide
MSI1: Multicopy suppressor of ira1
OAB: Outer apoplastic barrier
OTE: Oolong tea extract
PA: Proanthocyanidin
PcG: Polycomb group
SHN: SHINE
TEM: Transmission electron microscopy
TT: Transparent testa
VLCFA: Very-long-chain fatty acid
WBC11: White-brown complex homolog protein 11
WIN: Wax inducer
WRI: Wrinkled
