# Supplementary material for: Deposition of a cutin apoplastic barrier separating seed maternal and zygotic tissues
Source: BMC Plant Biol. 2019 Jul 10;19:304. doi: 10.1186/s12870-019-1877-9 (PMC6617593; doi:10.1186/s12870-019-1877-9)
Supplement: Supplementary file 1 — Figure S1. Detection of the IAB without staining. Figure S2. Detection of the IAB in different Arabidopsis ecotypes. Figure S3. The ovule IAB is made of nucellus and endothelium apoplastic barriers. Figure S4. Detection of the MAB in ovules. Figure S5. Expression of a set of genes involved in VLCFA deposition in ovules and seeds. Figure S6. IAB deposition in tt mutant seeds. Figure S7. tt16 seeds display an altered C18:2 DCA composition. Figure S8. tt16 and tt1 seeds exhibit altered expression of genes involved in VLCFA deposition. Figure S9. IAB deposition in mutant and over-expression lines of genes involved in cutin and wax deposition. Figure S10. IAB deposition is repressed by FIE and MSI1 FIS-PcG proteins. Table S1. Primers. Table S2. Solutions. Table S3. Student’s t test statistical analysis of GC-MS results. (PDF 1837 kb) [file 12870_2019_1877_MOESM1_ESM.pdf]

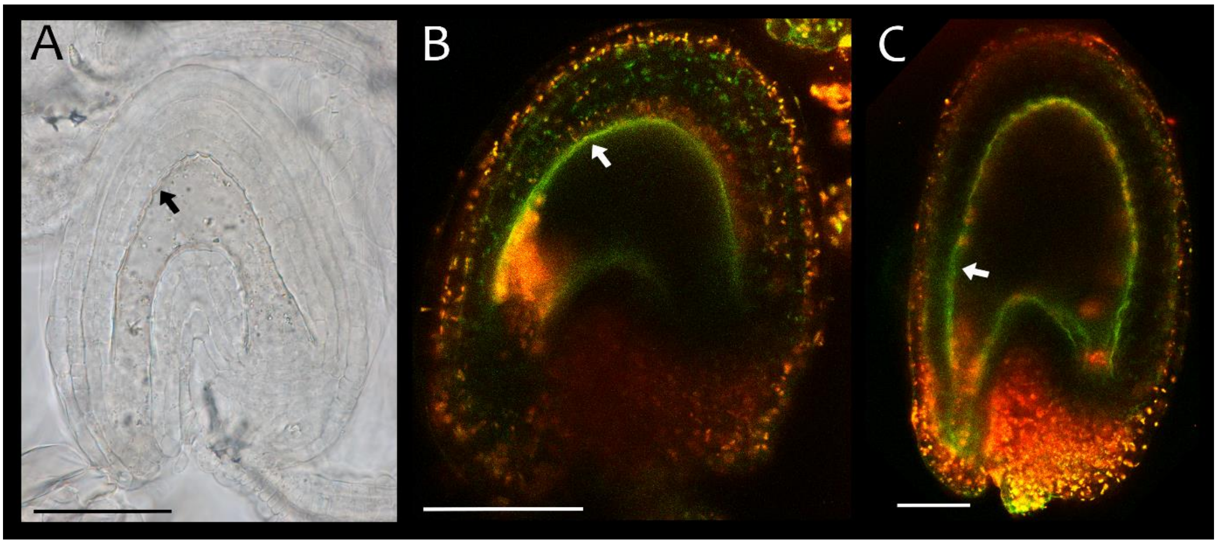

Additional file 1: Figure S1. Detection of the IAB without staining.
(A) Wild type seed analyzed using DIC microscopy.
(B) and **(C)** Auto-fluorescence images of wild type seeds. Excitation was performed at 488nm. Fluorescence was collected between 490nm and 530nm for the green channel and between 600nm and 700nm for the red channel.
Black and white arrows show the IAB. Ecotype Col. Scale bars: 50µm.

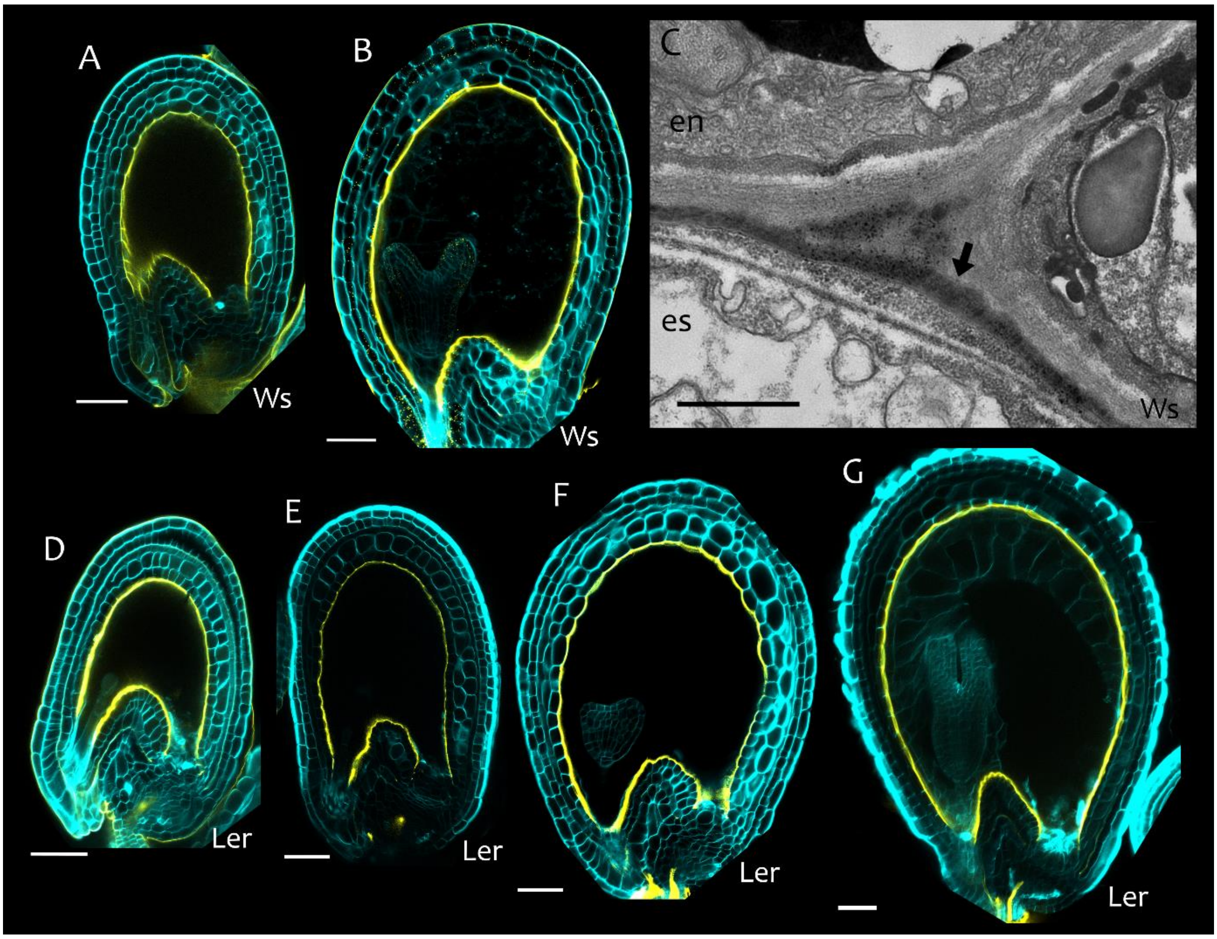

Figure S2. Detection of the IAB in different Arabidopsis ecotypes.
(A) to **(B)** and **(D)** to **(G)** Fluorescence images of longitudinal sections of seeds stained with auramine O (yellow) and counterstained with calcofluor (cyan). **(A)** to **(C)** Ecotype Ws.. **(D)** to **(G)** Ecotype Ler..
(C) Transmission electron micrograph showing the electron-dense IAB in a Ws seed at globular embryo stage of seed development. The black arrow indicates the IAB. en, endothelium; es, endosperm.
Scale bars: **(A)** to **(B)** and **(D)** to **(G)** 50µm, **(C)** 1µm.

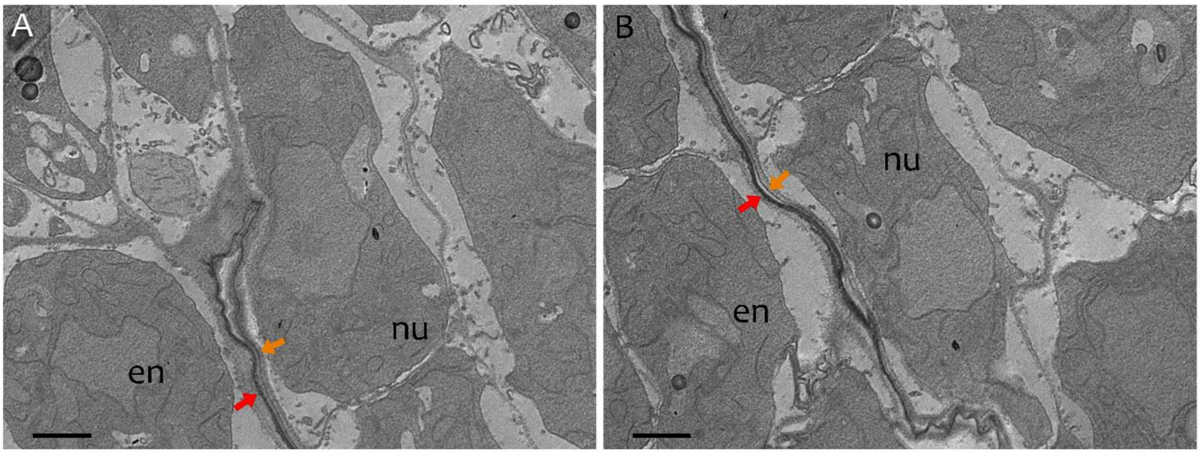

Figure S3. The ovule IAB is made of nucellus and endothelium apoplastic barriers.

(A) and **(B)** Transmission electron micrographs showing the electron-dense apoplastic barriers separating endothelium and nucellus in wild type ovules. **(A)** IAB proximal region. **(B)** IAB distal region. Red and orange arrows indicate the apoplastic barriers of the endothelium and the nucellus, respectively. en, endothelium; nu, nucellus. Scale bars: 1µm.

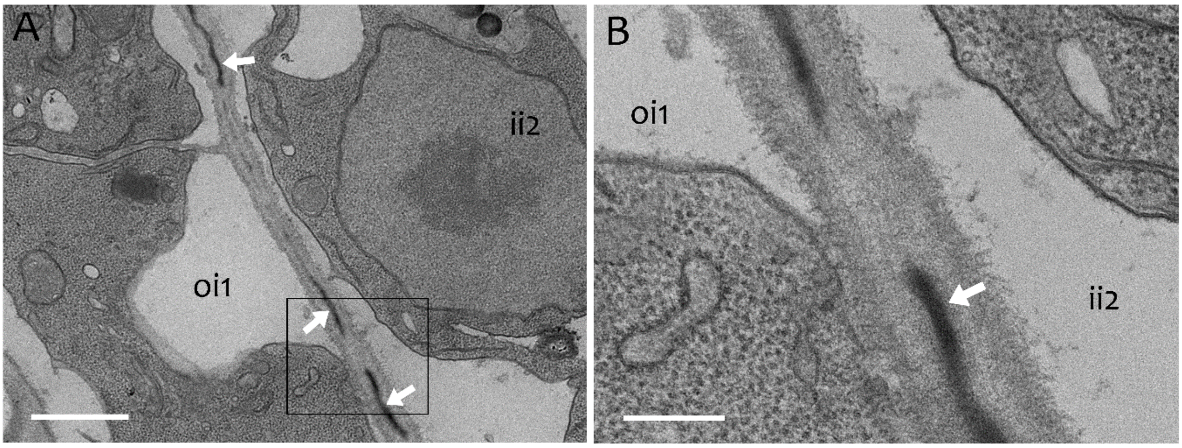

Figure S4. Detection of the MAB in ovules.

(A) Transmission electron micrographs showing the electron-dense MAB in a mature wild type ovule (stage 3-VI). **(B)** Close-up image of **(A)**. White arrows indicate the MAB. ii2, inner integument 2 cell layer; oi1, outer integument 1 cell layer. Scale bars: **(A)** 1µm, **(B)** 0,25µm.

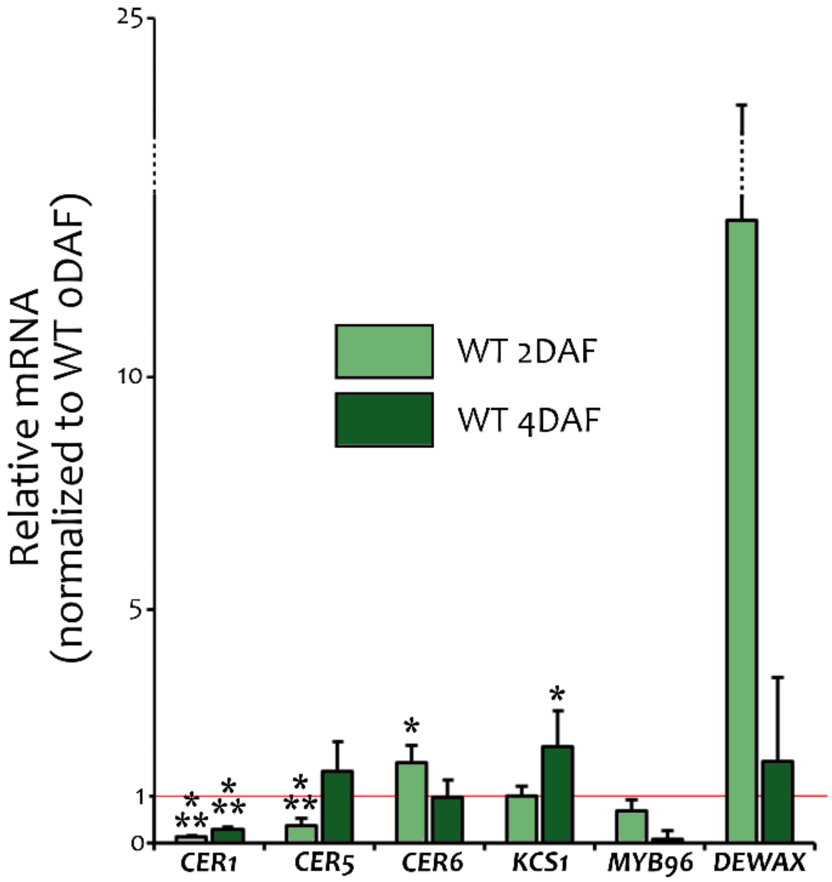

Figure S5. Expression of a set of genes involved in VLCFA deposition in ovules and seeds.

RT-qPCR analyses of genes involved in VLCFA deposition in wild type seeds at 2 and 4 DAF. Values are relative to wild type. Error bars represent standard deviations. Asterisks indicate statistical difference between different time points (Student's t test, *: P<0.05; **: P<0.01; ***: P<0.001). Ecotype Col.

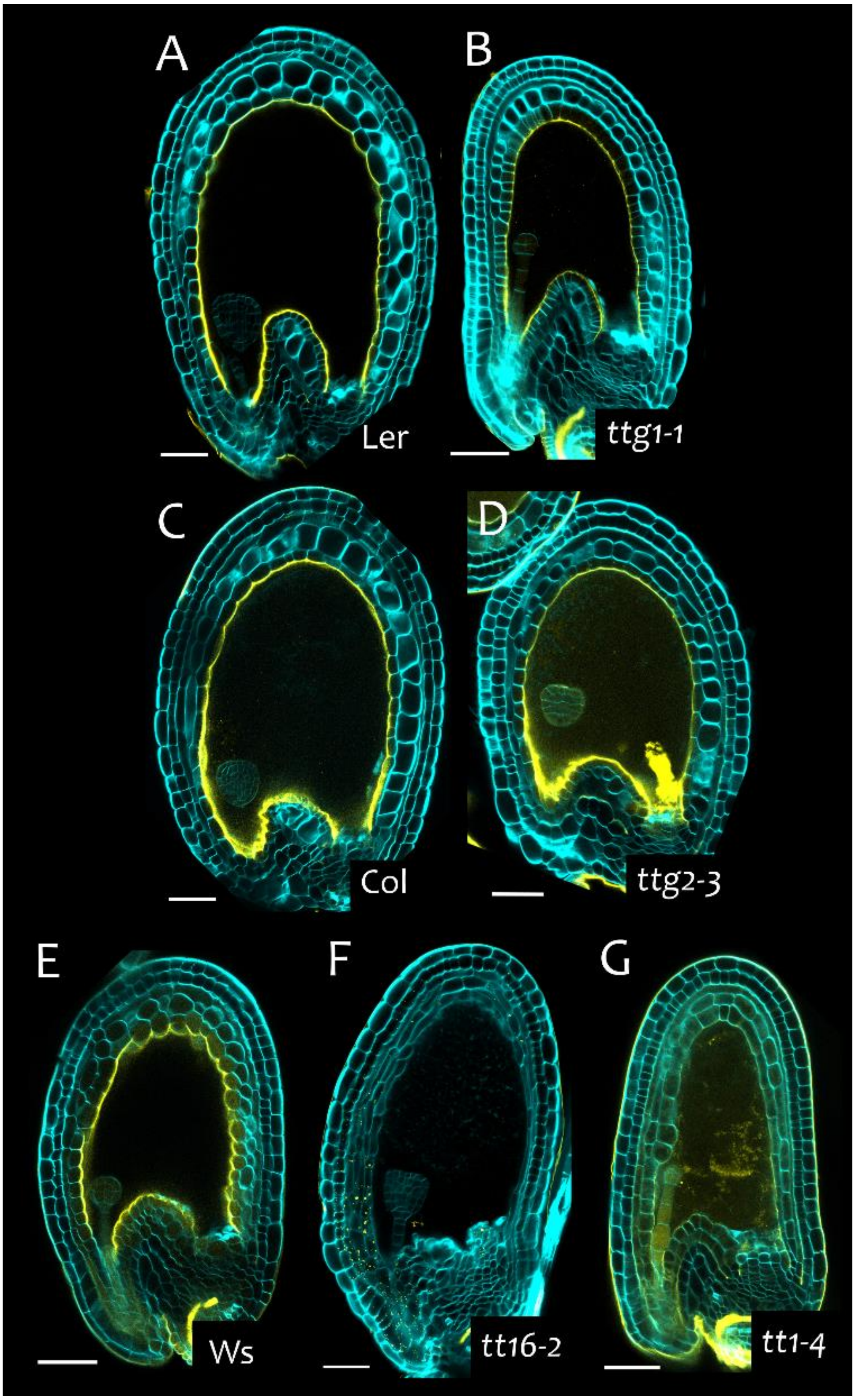

Figure S6. IAB deposition in *tt* mutant seeds.
(A) to **(G)** Fluorescence images of longitudinal sections of seeds stained with auramine O (yellow) and counterstained with calcofluor (cyan). **(A)** Wild type Ler. **(B)** *ttg1-1* (Ler). **(C)** Wild type Col. **(D)** *ttg2-3* (Col). **(E)** Wild type Ws. **(F)** *tt16-2* (Ws). **(G)** *tt1-4* (Ws). Scale bars: 50µm.

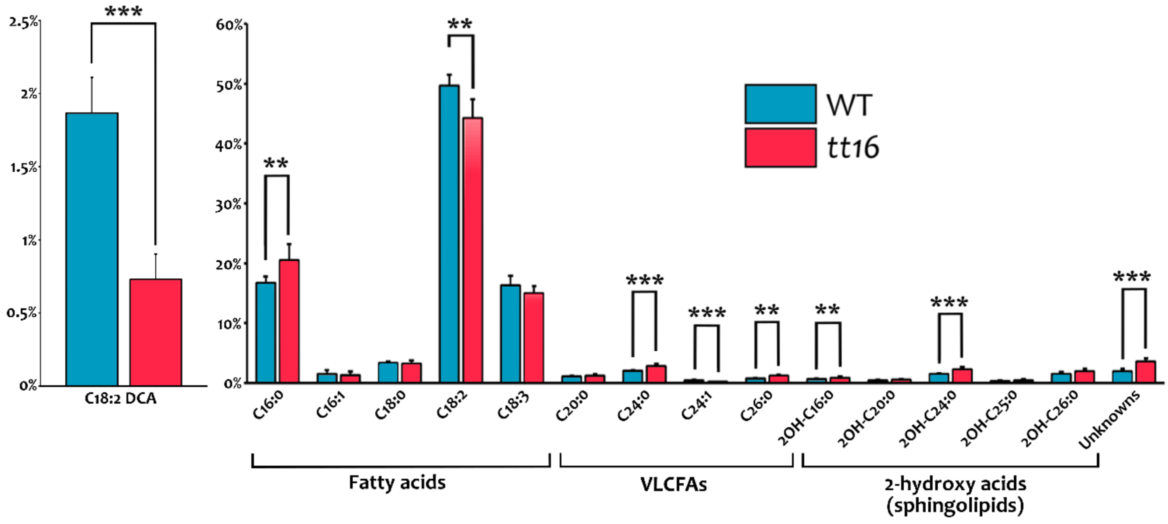

Figure S7. *tt16* seeds display an altered C18:2 DCA composition.
A biological replica of the GC-MS analysis of the fatty acyl composition of wild type and *tt16* seeds at 4 DAF in Figure 4. Values are relative to all detected fatty acyl chains. Error bars represent standard deviations. Asterisks indicate statistical difference between different genotypes (Student's t test, *: P<0.05; **: P<0.01; ***: P<0.001). Ecotype Col. The complete statistical analysis is in Additional Table 3.

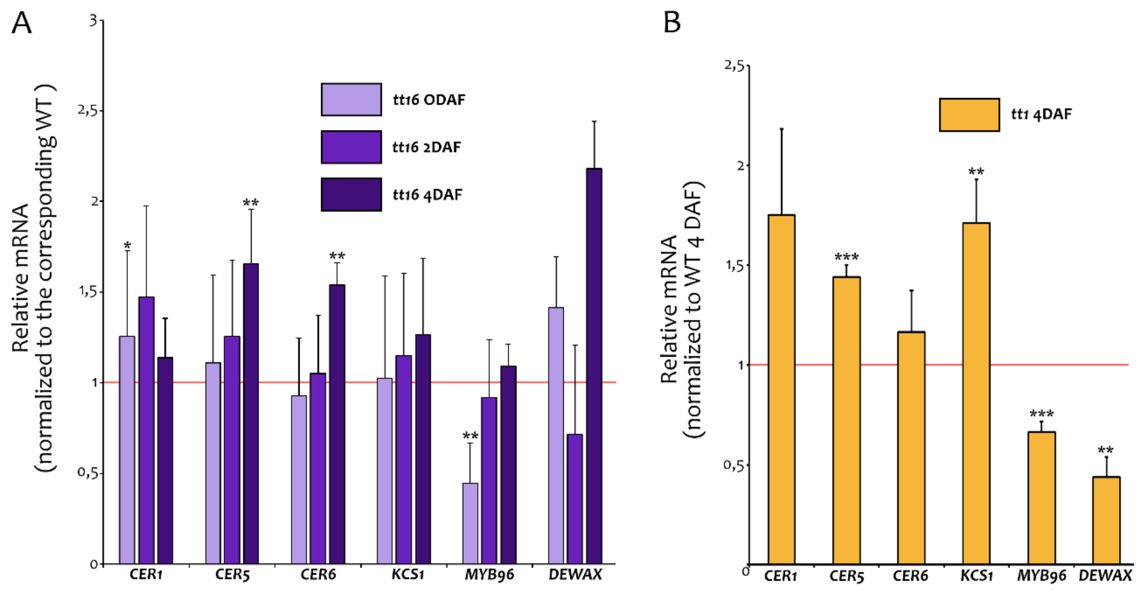

Figure S8. *tt16* and *tt1* seeds exhibit altered expression of genes involved in VLCFA deposition.

(A) and **(B)** RT-qPCR analyses of genes involved in VLCFA deposition in *tt16* ovules and seeds at 0, 2 and 4 DAF and in *tt1* seeds at 4DAF. Values are relative to wild type. Error bars represent standard deviations. Asterisks indicate statistical difference between mutant and wild type at the same time point (Student's t test, *: $P<0.05$; **: $P<0.01$; ***: $P<0.001$). Ecotype Col.

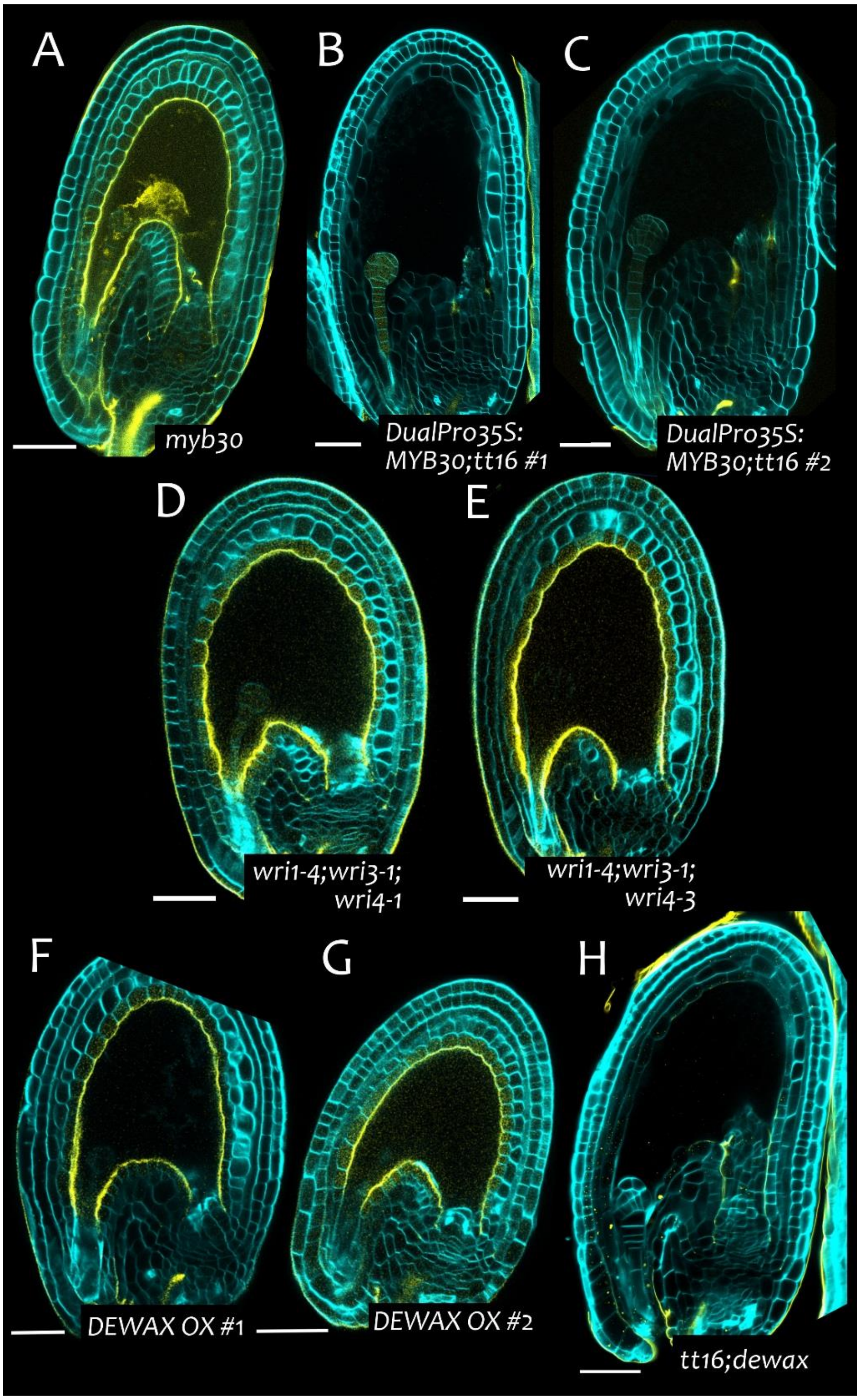

Figure S9. IAB deposition in mutant and over-expression lines of genes involved in cutin and wax deposition.
(A) to **(H)** Fluorescence images of longitudinal sections of seeds stained with auramine O (yellow) and counterstained with calcofluor (cyan). **(A)** *myb30* mutant seed. **(B)** and **(C)** Seeds from two independent *Pro35S:cMYB30;tt16* lines. **(D)** *wri1-4;wri3-1;wri4-1* triple mutant seed. **(E)** *wri1-4;wri3-1;wri4-3* triple mutant seed. **(F)** and **(G)** Seeds from two independent *Pro35S:cDEWAX* lines. **(H)** *tt16;dewax* double mutant seed. Scale bars: 50µm.

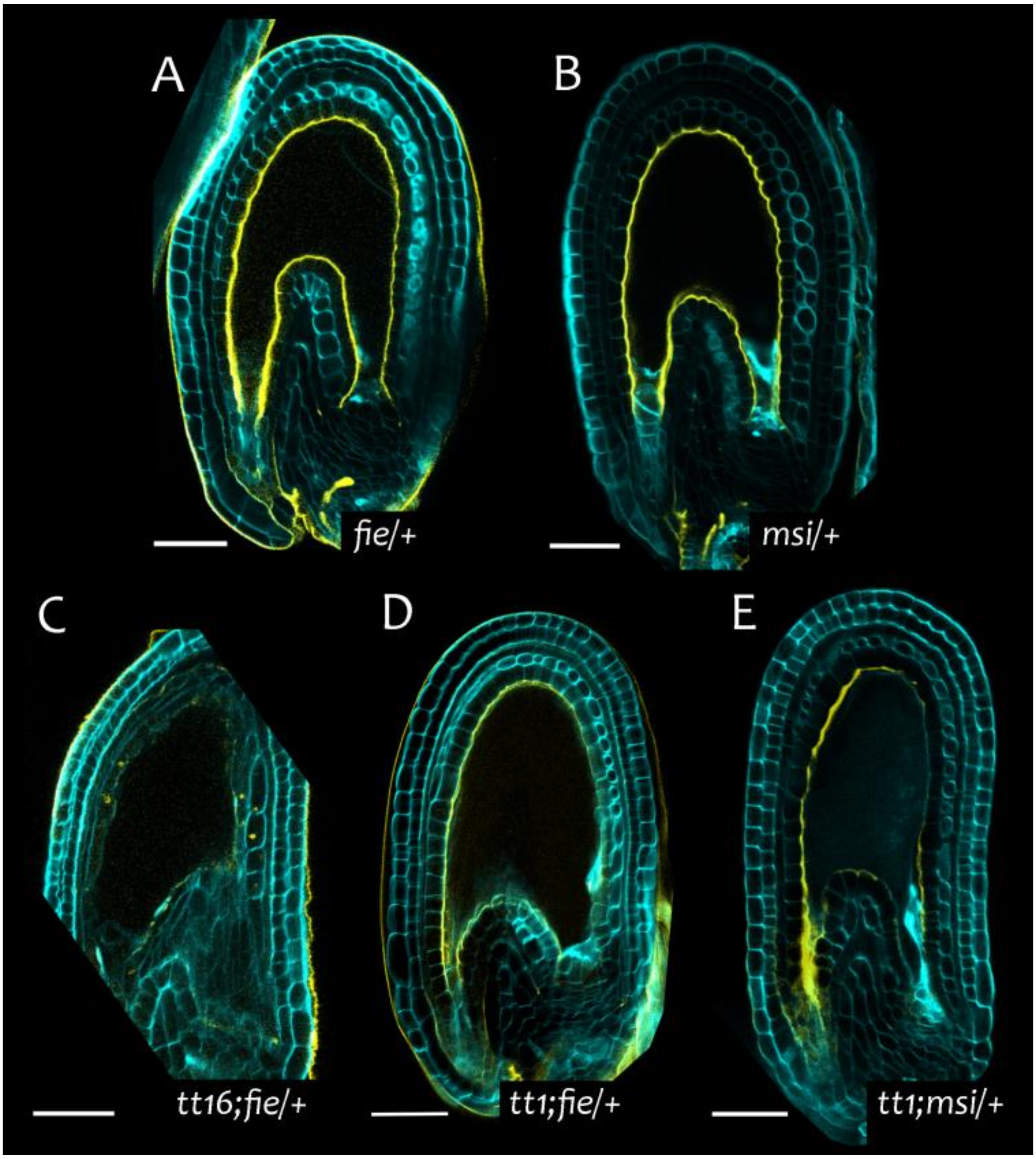

Figure S10. IAB deposition is repressed by FIE and MSI1 FIS-PcG proteins.

(A) to **(E)** Fluorescence images of longitudinal sections of large autonomously-developed seeds at 6DAE. **(A)** *fie/+*. **(B)** *msi1/+*. **(C)** *tt16;fie/+*. **(D)** *tt1;fie/+*. **(E)** *tt1;msi1/+*. Scale bars: 50µm.

Table S1. Primers

Cloning

pTT1 in *pGWB2*

pGWB2-pTT1-F	TGATTACGCCAAGCTATACAGTATATTAGAAGTAATACTTG
pGWB2-TT1bfATG-R	TGTTGATAACTCTAGTGAATGTGGTGAATAGTTGTTGG

NTF in *pDONR207*

B1-NTF-F	GGGGACAAGTTTGTACAAAAAAGCAGGCTCTATGGATCATTCAGCGAAAACCACAC
B2-NTF-R	GGGGACCACTTTGTACAAGAAAGCTGGGTCTCAAGATCCACCAGTATCCTCATGC

cMYB30 in *pDONR207*

B1-cMYB30-F	GGGGACAAGTTTGTACAAAAAAGCAGGCTATGGTGAGGCCTCCTTGTTGT
B2-cMYB30-R	GGGGACCACTTTGTACAAGAAAGCTGGGTCTCAGAAGAAATTAGTGTTTTCATCC

pMYB30:gMYB30 in *pDONR207*

B2-pMYB30-F2	GGGGACCACTTTGTACAAGAAAGCTGGGTCGAAGAAATTAGTGTTTTCATCC
B2-MYB30nostop-R	GGGGACCACTTTGTACAAGAAAGCTGGGTCGAAGAAATTAGTGTTTTCATCCAATAG

RT-qPCR

Reference genes

GAPDH-Q-F	GGTACGACAACGAATGGGGT	GAPDH-Q-R	TGACTGCGCATGGAATCAGT
AT3G25800-Q-F	AATCGGTTGTGGAGAAGACG	AT3G25800-Q-R	GCGAAAAACCTGACATCAACAT
AT4G02080-Q-F	GCTGTGTTATTATTAAGCCGTAAG	AT4G02080-Q-R	AAAGCTAGGTACGGTTTAAGAC
AT4G12590-Q-F	GAGATGAAAATGCCATTGATGAC	AT4G12590-Q-R	GCACCCAGACTCTTTGATG

Target genes

ATT1-Q-F	CGTGTTCCATGACTTCCTCG	ATT1-Q-R	AGAATTGGACAAAACCGGAGC
BDG-Q-F	GTTAAACACCCAGGAGCCATC	BDG-Q-R	GACTGACTGTGCGGCTAATG
DCR-Q-F	AGCCCAATCCATCTCGACTC	DCR-Q-R	CGCCAGATGTGTGATGTCAG
GPAT4-Q-F	TCATCTCCTTCGCCGGTATC	GPAT4-Q-R	CGCAGTCACCACTACTTTCC
FATB-Q-F	CGTTCTGACATGCTGGTGG	FATB-Q-R	AACCCATCTCCAAGCAATCC
LACS2-Q-F	GCAGCAATTTCGGTCCAGG	LACS2-Q-R	ACGCCAGTATCCAACAGAGG
WBC11-Q-F	GCAAGCAGACAACAAACCAC	WBC11-Q-R	CACCGTCAGATCTTGCCAC
WRI1-Q-F2	ACAACAATGAGCTGGCTTGG	WRI1-Q-R2	AAGAAGAAGAGGGTGGGCTC
WRI3-Q-F2	GCCGTTATCTGAAACTCCCG	WRI3-Q-R2	ACCTCCTCTGCCACTAAAGG

WRI4-Q-F3	GGCAAAAGTCTCTGGGAGGA	WRI4-Q-R3	CGTTGAAGAGGAGCGTTTCG
CER1-Q-F	GCTACCATTCCCACCACCAC	CER1-Q-R	AGTTGCAGTGTCCCATGTTG
CER5-Q-F	GACACCAGCTACATCAGATCC	CER5-Q-R	AGCTGCTTAAACCACGTCG
CER6-Q-F	CCCTCAAGGCAAACATCACC	CER6-Q-R	GGACGTGAGGAAGAGAAGTTG
KCS1-Q-F	GTTTTGACCCTCTACGTGGC	KCS1-Q-R	TTTGCTGGAACTGAACCGTG
MYB30-Q-F4	CGCTCTCATCTTCACCATCG	MYB30-Q-R4	TCAGCAGAGGAAGACGTTGT
MYB96-Q-F2	CCACAACCACCACTACAAGC	MYB96-Q-R2	TCCACCTTCTTCCGAGACTG
DEWAX-Q-F	CCGAAACTGGAACCTAGTTCA	DEWAX-Q-R	TTCTTTGCCGGATCTCGAATC

RNA *in situ* hybridization

AntiTT1-F	ATGGAGTCACCACCACTATA	T7-AntiTT1-R	TGTAATACGACTCACTATAGGGCGCATA CATGGCAAGAAAAAT
AntiHis4-F	CATCTCAATCTCAATTAAATCTT	T7-AntiHis4-R	TGTAATACGACTCACTATAGGGCATACTA AACAAGCATCGAGAAACT

Table S2. Solutions

**GUS staining**
**Solution n°1**
0.052g of X-Gluc in 500 µL of dimethylformamide,<br>diluted in 50 mL of $NaH_2\ PO_4$ 28 mM, Na_2HPO_4 72 mM, EDTA 10mM pH 7,5, Triton 0.1X
**TEM preparation**
**Solution n°2 (Fixation)**<br>Glutaraldehyde (Sigma, 2%), fresh formaldehyde (Sigma, 0.5%) in cacodylate buffer 0.1 M at pH 7
**RNA *in situ* hybridization**
**Solution n°3 (Fixation)**<br>Fresh formaldehyde (Sigma, 4%), Triton (1%) in PBS 1X buffer
**Solution n°4 (Proteinase K solution)**<br>Proteinase K (Sigma, 1 mg L^{-1}) in Tris (100 mM, pH 7,5) EDTA (50 mM) buffer
**Solution n°5 (Acetic anhydride / triethanolamine-HCl)**<br>Triethanolamine (1.5%) in water (pH adjusted at 8 with HCl), 0.5% acetic anhydride
**Solution n°6 (Prehybridization buffer)**<br>De-ionised formamide (50%), 5X SSC, heparin (50 µg mL^{-1}), tRNA (Roche, 100 µg mL^{-1}), Tween (0.02%)
**Solution n°7 (Hybridization buffer)**<br>*TT1* RNA probe (0.4 ng μL^{-1}), de-ionised formamide (50%), Dextran Sulfate (Sigma, 20%), tRNA (Roche, 1 mg mL^{-1}), Tween (0.15%), Ficoll (0.05%), PVP (0.05%), BSA (0.05%), NACL (0.3 M), Tris pH 8 (10mM), EDTA (1 mM)
**Solution n°8 (RNAse solution)**<br>RNAse (Roche, 20 µg mL^{-1}) in NTE
**Solution n°9 (Wash buffer n°1 before detection)**<br>Tris pH 7.5 (100 mM)**,** NaCl (150 mM), Blocking reagent (Roche, 0.5 %)
**Solution n°10 (Wash buffer n°2 before detection)**<br>Tris pH 7.5 (100 mM)**,** NaCl (150 mM), BSA (Sigma, 1%), Triton 0.5X
**Solution n°11 (Detection)**<br>Digoxygenin-targeting antibody (Roche), dilution 1/1250 in solution n°10
**Solution n°12 (Washing and staining buffer)**<br>Tris pH 9,5 (100 mM), NaCl (100 mM), $MgCl_2$ (50 mM)
**Solution n°13 (Staining)**<br>NBT (Nitroblue tetrazolium chloride, Roche, 337.5 µg mL^{-1}), XP (5-bromo-4-chloro-3-indolyl-phosphate, Roche, 175 µg mL^{-1}) in solution n°12

Table S3. Student's t test statistical analysis of GC-MS results.

GC-MS analysis in Figure 2-A

	4 DAF vs 0 DAF	8 DAF vs 4 DAF	8 DAF vs 0 DAF
C16:0	4,05E-01	2,49E-01	7,60E-02
C16:1	8,10E-01	**2,03E-02**	5,16E-02
C16:3	6,06E-01	7,35E-01	1,28E-01
C18:0	**4,75E-04**	**8,71E-05**	**2,89E-03**
C18:2	**2,61E-02**	**1,39E-02**	**4,62E-01**
C18:3	1,87E-01	9,18E-01	4,37E-01
C20:0	**8,18E-04**	**3,55E-03**	3,33E-01
C22:0	1,00E-01	2,45E-01	7,54E-01
C23:0	**3,34E-03**	**1,18E-02**	7,07E-02
C24:0	4,12E-01	8,92E-01	5,09E-01
C24:1	**1,61E-02**	9,86E-01	4,38E-01
C25:0	**3,08E-03**	**2,79E-03**	**3,88E-02**
C26:0	5,20E-01	**3,19E-02**	8,75E-02
C18:2 DCA	**2,66E-02**	**1,70E-02**	**2,09E-02**
2OH-C16	1,31E-01	**1,04E-02**	1,08E-01
2OH-C22	**6,32E-03**	**2,92E-04**	**1,80E-03**
2OH-C24:0	**1,64E-02**	**1,42E-02**	**5,63E-03**
2OH-C24:1	1,62E-01	**7,37E-04**	**7,72E-03**
2OH-C25:0	1,07E-01	1,95E-01	4,53E-01
2OH-C26:0	1,45E-01	9,60E-01	7,49E-01
Unknown	**4,39E-01**	**1,38E-03**	**1,60E-01**

GC-MS analysis in Figure 4

	WT vs *tt16*	WT vs *tt1*
C16:0	4,70E-01	8,96E-01
C16:1	3,50E-01	**4,02E-01**
C16:3	9,34E-01	7,99E-01
C18:0	**5,76E-02**	**3,38E-03**
C18:2	**4,90E-02**	**5,63E-02**
C18:3	1,12E-01	1,77E-01
C20:0	**3,53E-03**	**3,47E-01**
C22:0	4,41E-01	9,93E-01
C23:0	**1,21E-02**	**9,92E-02**
C24:0	1,45E-01	5,71E-01
C24:1	**3,78E-04**	5,12E-04
C25:0	**3,11E-02**	**2,57E-01**
C26:0	4,73E-01	**2,04E-01**
C18:2 DCA	**2,75E-03**	**1,83E-03**
2OH-C16	8,40E-02	**7,87E-02**
2OH-C22	**8,89E-01**	**3,20E-01**

**2OH-C24:0**	**3,38E-01**	**7,53E-01**
**2OH-C24:1**	4,26E-01	**1,25E-01**
**2OH-C25:0**	5,39E-02	3,90E-01
**2OH-C26:0**	9,63E-01	1,19E-01
**Unknown**	**4,05E-01**	**3,26E-01**

GC-MS analysis in Additional Figure 6

	**WT vs *tt16***
**C16:0**	9,30E-03
**C16:1**	7,43E-01
**C18:0**	**6,68E-01**
**C18:2**	**4,19E-03**
**C18:3**	1,43E-01
**C20:0**	**4,33E-01**
**C24:0**	2,64E-04
**C24:1**	**2,29E-04**
**C26:0**	5,06E-03
**C18:2 DCA**	**2,84E-06**
**2OH-C16**	1,27E-03
**2OH-C22**	**1,44E-01**
**2OH-C24:0**	**7,70E-04**
**2OH-C25:0**	1,17E-01
**2OH-C26:0**	9,98E-02
**Unknowns**	**1,99E-04**
